# Structural mechanism for gating of a eukaryotic mechanosensitive channel of small conductance

**DOI:** 10.1038/s41467-020-17538-1

**Published:** 2020-07-23

**Authors:** Zengqin Deng, Grigory Maksaev, Angela M. Schlegel, Jingying Zhang, Michael Rau, James A. J. Fitzpatrick, Elizabeth S. Haswell, Peng Yuan

**Affiliations:** 1 Department of Cell Biology and Physiology, Washington University School of Medicine, Saint Louis, MO 63110 USA; 2 Center for the Investigation of Membrane Excitability Diseases, Washington University School of Medicine, Saint Louis, MO 63110 USA; 3 Department of Biology, Washington University in Saint Louis, Saint Louis, MO 63130 USA; 4 NSF Center for Engineering Mechanobiology, Washington University in Saint Louis, Saint Louis, MO 63130 USA; 5 Washington University Center for Cellular Imaging, Washington University School of Medicine, Saint Louis, MO 63110 USA; 6 Department of Neuroscience, Washington University School of Medicine, Saint Louis, MO 63110 USA; 7 Department of Biomedical Engineering, Washington University in Saint Louis, Saint Louis, MO 63130 USA

**Keywords:** Cryoelectron microscopy, Ion transport

## Abstract

Mechanosensitive ion channels transduce physical force into electrochemical signaling that underlies an array of fundamental physiological processes, including hearing, touch, proprioception, osmoregulation, and morphogenesis. The mechanosensitive channels of small conductance (MscS) constitute a remarkably diverse superfamily of channels critical for management of osmotic pressure. Here, we present cryo-electron microscopy structures of a MscS homolog from *Arabidopsis thaliana*, MSL1, presumably in both the closed and open states. The heptameric MSL1 channel contains an unusual bowl-shaped transmembrane region, which is reminiscent of the evolutionarily and architecturally unrelated mechanosensitive Piezo channels. Upon channel opening, the curved transmembrane domain of MSL1 flattens and expands. Our structures, in combination with functional analyses, delineate a structural mechanism by which mechanosensitive channels open under increased membrane tension. Further, the shared structural feature between unrelated channels suggests the possibility of a unified mechanical gating mechanism stemming from membrane deformation induced by a non-planar transmembrane domain.

## Abstract (web summary)

Mechanosensitive channels transduce physical force into electrochemical signaling in processes such as hearing, touch, proprioception, osmoregulation, and morphogenesis. Here, authors use cryo-electron microscopy to provide structural insights into the mechanical gating mechanism.

## Introduction

Mechanical force sensation mediated by mechanosensitive (MS) ion channels represents a prevalent and fundamental biological process essential for all kingdoms of life^1–3^. MS channels underlie osmoregulation in bacteria, hearing, touch, and proprioception in animals, and are proposed to underlie response to osmotic stress, touch, vibration, and gravity and developmental signals in plants^4–11^. The prokaryotic MS channel of small conductance (MscS) opens in response to hypoosmotic downshock, allowing rapid efflux of solvent and solutes and thereby protecting bacterial cells from rupture^12–14^. Found in many organisms including bacteria, fungi, algae, and plants, MscS-Like (MSL) channels form a remarkably diverse superfamily of MS channels that appear to be pivotal for management of osmotic pressure^3^.

Intrinsically sensitive to membrane tension, MscS channels from several prokaryotes have been extensively characterized and served as a prevailing model system for understanding physicochemical principles in mechanotransduction^9^. X-ray and cryo-electron microscopy (Cryo-EM) structures reveal that each protomer of the homo-heptameric MscS channel consists of three transmembrane helices (TM1-3) followed by a cytoplasmic barrel structure^15–22^. The presumed closed and open structures suggest that TM1 and TM2 constitute a peripheral membrane ‘tension sensor’, which is attached to the central pore-lining helix TM3a followed by the amphipathic TM3b running approximately parallel to the membrane^15–18^. According to this model, rotation and tilting of TM1 and TM2 as a rigid body under elevated membrane tension, accompanied by displacement of channel-bound lipid molecules, pulls TM3a to open the hydrophobic pore gate^16,18^. However, different gating models derived from electron paramagnetic resonance (EPR) spectroscopy, molecular dynamics (MD) simulations, and recent cryo-EM studies have also been proposed^20,23–26^, and these competing models are actively debated^9,20^.

Much less is known about the structure of eukaryotic members of the MscS superfamily. With only limited homology in the pore-lining helix and the subsequent cytosolic portion, many acquire extra transmembrane helices as well as additional extramembrane domains^3^. These added structural elements may give rise to rich channel properties^27^, which have probably evolved to fulfill adapted functionalities in particular physiological settings. Fundamental questions naturally arise. How are these extra transmembrane helices organized in the membrane? Do they sense mechanical stimulation and contribute to channel gating?

Ten MSL channels have been identified in the land plant *A. thaliana*, and they exhibit distinct membrane topology, domain organization, cellular localization, and physiological functions^3^. MSL1, predicted to contain five transmembrane helices and localized to the inner membranes of mitochondria, is involved in the regulation of membrane potential and maintenance of redox homeostasis under abiotic stress^28^. When heterologously expressed in giant *E. coli* spheroplasts, MSL1 displays stretch-activated gating in excised patches and channel properties similar to those of *E. coli* MscS (*Ec*MscS)^28^, which demonstrates that MSL1 forms a functional mechanosensitive channel. To gain insights into how mechanosensitive channels gate, we have determined structures of *A. thaliana* MSL1 (*At*MSL1), presumably representing the closed and open conformations, by single-particle cryo-EM. Our structures, in combination with electrophysiology experiments, reveal a structural mechanism by which (at least some) mechanosensitive channels open in response to increased membrane tension.

## Results

### Structure determination of the AtMSL1 channel

**Fig. 1:**
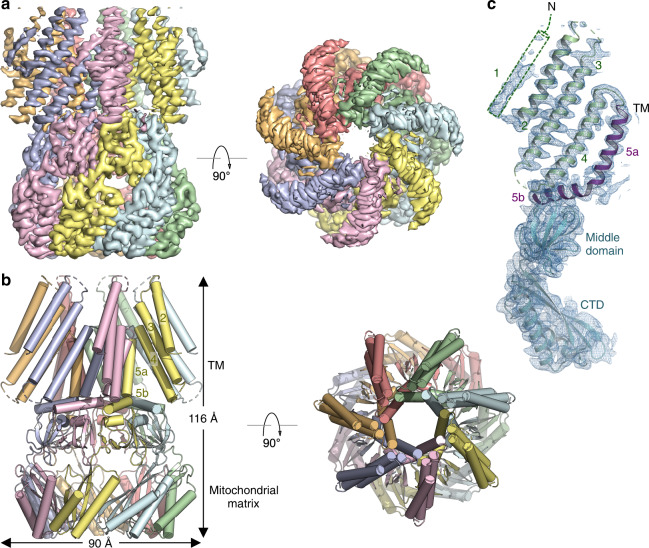
Cryo-EM structure of *At*MSL1. **a** Orthogonal views of the cryo-EM density contoured at 7.0 σ. Each subunit is uniquely colored. **b** Orthogonal views of the MSL1 structure. Transmembrane helices TM2-5, the C-terminal extramembrane domain located in the mitochondrial matrix, and the dimension of the channel are indicated. **c** A single subunit of MSL1 is shown in cartoon representation with TM helices (TM2-4 in green and TM5 in purple) and extramembrane domains (cyan) indicated. Cryo-EM density contoured at 6.5 σ is shown in blue mesh.

We expressed an *At*MSL1 construct consisting of amino acids 80–497, which lacks the mitochondrial targeting sequence, in the yeast *P. pastoris* and determined the cryo-EM structure at an overall resolution of ~3.1 Å (Fig. 1a–c, Supplementary Figs. 1, 2, Table 1). The overall architecture and domain organization of the heptameric *At*MSL1 channel resemble those of the *Ec*MscS channel (Supplementary Figs. 3 and 4). In contrast to *Ec*MscS, which comprises three membrane-spanning helices (TM1-3), *At*MSL1 indeed possesses two additional N-terminal transmembrane helices, resulting in a transmembrane domain with five membrane-spanning helices (TM1-5). Akin to TM3 in *Ec*MscS, the innermost helix TM5 is kinked such that TM5a lines the pore and TM5b runs tangentially to the central pore axis and connects to the middle and C-terminal domains, which are located in the mitochondrial matrix (Fig. 1c). The transmembrane domain is assembled as a seven-bladed propeller, in which each blade comprises peripheral transmembrane helices (TM1-4) organized approximately in a straight line surrounding the pore-lining helix TM5. This unusual structural arrangement creates large unoccupied spaces between the blades, which would presumably be filled with lipids in a membrane environment (Fig. 1b). Probably owing to limited protein-protein contacts between blades, the peripheral transmembrane helices TM1-4 are less well resolved than the remaining part of the channel in the cryo-EM density map (Fig. 1a, Supplementary Fig. 2). Density for the first N-terminal transmembrane helix TM1, which is located at the outer perimeter, is present in the cryo-EM map but rather weak, and thus TM1 is not modeled in the structure (Fig. 1c). The middle and C-terminal domains of *At*MSL1, including the extramembrane side portals, are analogous to those of *Ec*MscS except that *At*MSL1 lacks the C-terminal seven-stranded β-barrel present at the extreme C-terminus of *Ec*MscS (Supplementary Fig. 4).

**Table 1:** Cryo-EM data collection, refinement and validation statistics.

	*At*MSL1 in detergents (EMDB: 21444) (PDB: 6VXM)	*At*MSL1 A320V in detergents (EMDB: 21445) (PDB: 6VXN)	*At*MSL1 in nanodiscs (EMDB: 21447) (PDB: 6VXP)
*Data collection and processing*			
Magnification	105,000	105,000	105,000
Voltage (kV)	300	300	300
Electron exposure (e–/Å^2^)	62	62	62
Defocus range (μm)	−1.0 to −2.5	−1.0 to −2.5	−1.0 to −2.5
Pixel size (Å)	1.1	1.1	1.1
Symmetry imposed	C7	C7	C7
Initial particle images (no.)	936,130	428,889	457,683
Final particle images (no.)	158,172	52,883	23,077
Map resolution (Å)	3.06	2.96	3.39
FSC threshold	0.143	0.143	0.143
Map resolution range (Å)	2.5–6.0	2.5–6.0	3.0–7.0
*Refinement*			
Initial model used (PDB code)	5AJI	This study	This study
Model resolution (Å)	3.0	3.1	3.5
FSC threshold	0.5	0.5	0.5
Model resolution range (Å)	2.7	2.7	3.1
Model composition			
Non-hydrogen atoms	15,064	14,385	14,819
Protein residues	1939	1862	1939
Ligands	21	7	0
*B* factors (Å^2^)			
Protein	36.2	93.1	73.6
Ligand	20	111	N/A
R.m.s. deviations			
Bond lengths (Å)	0.006	0.008	0.008
Bond angles (°)	0.763	1.283	1.126
Validation			
MolProbity score	1.57	1.28	1.64
Clashscore	5.58	5.31	6.59
Poor rotamers (%)	0	0	0
Ramachandran plot			
Favored (%)	96.1	98.45	95.94
Allowed (%)	3.9	1.55	4.06
Disallowed (%)	0	0	0

Our structure is further corroborated by a recent independent cryo-EM structure of *At*MSL1 that was expressed in mammalian cells^29^. The root-mean-square deviation (r.m.s.d) between these two structures is ~1.4 Å for Cα atoms from TM4 to the C-terminus. The most pronounced differences arise from the peripheral TM2 and TM3 helices that appear to be dynamic owing to lack of protein-protein interactions within the heptameric channel (r.m.s.d ~3.1 Å for Cα atoms in TM2 and TM3).

### Bowl-shaped transmembrane domain

**Fig. 2:**
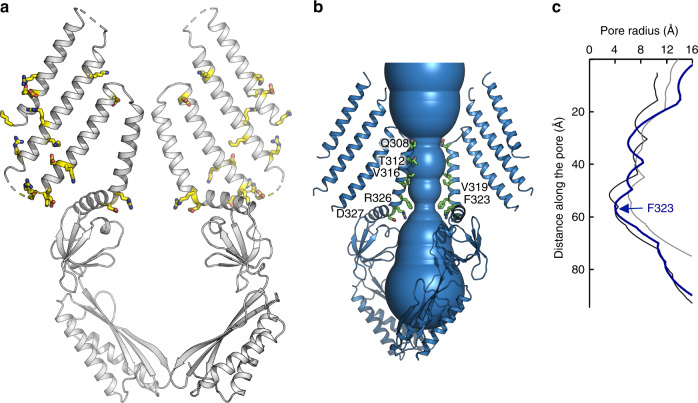
The closed conformation of *At*MSL1. **a** Distribution of charged residues, shown in stick representation, in the transmembrane helices indicates a curved transmembrane region. Only two opposing MSL1 subunits are shown for clarity. **b** The pore structure. Residues lining the pore are highlighted in stick representation. **c** The pore profile of MSL1 (blue). Also shown are the pore profiles of the closed (black, PDB: 6RLD) and open (gray, PDB: 2VV5) *Ec*MscS structures.

The bowl-shaped transmembrane domain of *At*MSL1 suggests that it naturally resides in locally curved, rather than planar, biological membranes (Fig. 2a). This notion is further supported by the distribution of charged residues in these transmembrane helices, which presumably demarcate the boundary of lipid bilayers (Fig. 2a). The cryo-EM structure was determined in detergent micelles that are virtually devoid of lipids. Thus, to assess whether this unusual shape is an intrinsic property of *At*MSL1, rather than an artifact owing to the detergent environment, we also determined the cryo-EM structure of *At*MSL1 embedded into lipid nanodiscs, which closely mimic biological membranes^30^, at a lower resolution of 3.4 Å (Supplementary Fig. 5). *At*MSL1 structures in nanodiscs and detergents are essentially identical (r.m.s.d of ~0.1 Å for all Cα atoms), demonstrating that the curved transmembrane region is inherent to *At*MSL1. In line with our structures, a low-resolution (~13 Å) cryo-EM map of the bacterial MscS homolog YnaI, which also contains five TM helices, hinted at a curved membrane surrounding its tapered transmembrane domain^31^. Furthermore, our *At*MSL1 structures are reminiscent of the eukaryotic mechanosensitive Piezo channels, which contain extended arms that are apparently curved within a membrane^32–34^. This shared structural feature between evolutionarily and architecturally unrelated MS channels points to the possibility of a unified mechanical gating mechanism stemming from membrane deformation induced by a non-planar transmembrane domain^32^.

In *Ec*MscS, two rings of bulky hydrophobic side chains from L105 and L109 create a ‘vapor lock’ that blocks ion passage by dewetting the channel pore^35^. The corresponding residues in *At*MSL1, V319 and F323, likely play an equivalent role in forming a hydrophobic gate (Fig. 2b, c). Pore radius calculation reveals that F323, near the C-terminal end of the pore-lining helix TM5a, defines the narrowest constriction along the central permeation pathway, which is comparable to the closed *Ec*MscS gate in dimension. This indicates that the *At*MSL1 structure represents a non-conducting, resting state, which is consistent with the absence of membrane tension in detergent micelles and lipid nanodiscs where the structures were determined.

### Open conformation of AtMSL1

**Fig. 3:**
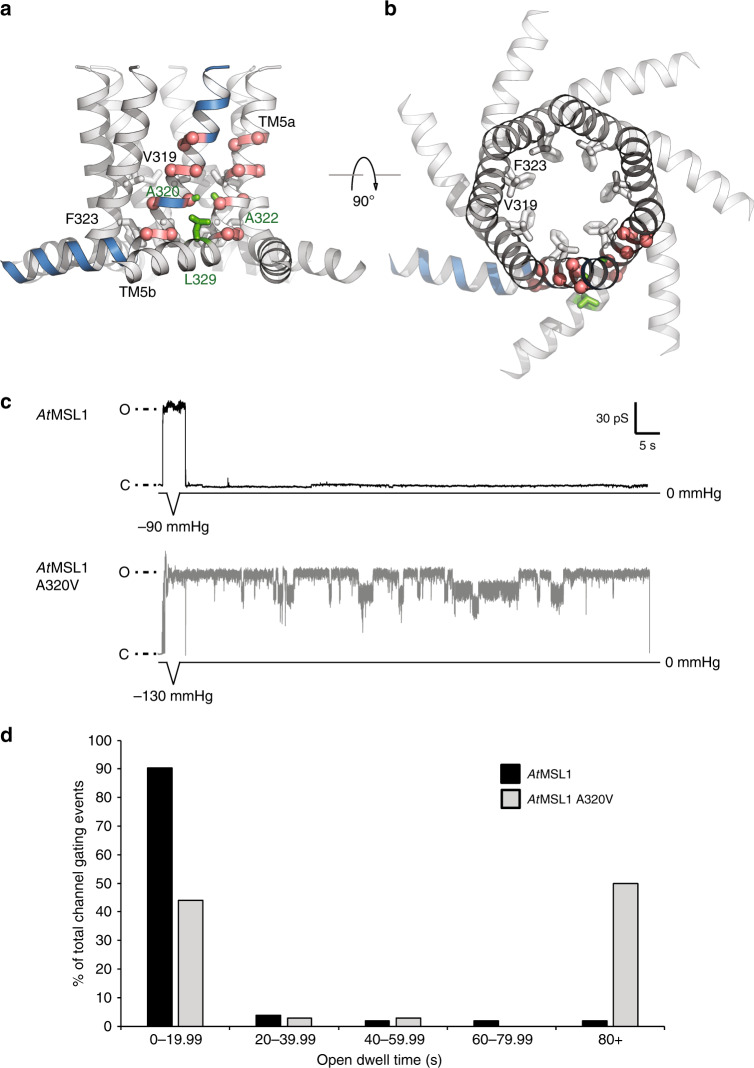
Helix-helix interface and the A320V mutant. **a**, **b** Orthogonal views of TM5. Glycine and alanine residues are indicated as red spheres. A320 and its contacts in a neighboring helix (A322 and L329) are highlighted in green. V319 and F323 constitute the hydrophobic gate. **c** Representative traces for single *At*MSL1 and A320V channel activities in response to a brief 1 s pressure ramp followed by 90 s without pressure. Closed state (baseline) current is indicated by C while open states are indicated by O. The maximum negative pressure used is indicated. Membrane potential was clamped at −70 mV. Multiple subconducting states were observed with the A320V mutant, while the peak unitary current of A320V was indistinguishable from that of the wild type (Table 3). **d** Percentages of *At*MSL1 (black) and *At*MSL1 A320V (gray) gating events with open dwell times of 0–19.99 s, 20–39.99 s, 40–59.99 s, 60–79.99 s, or 80+ s. Data represent traces obtained from 10 patches per variant channel with only the first gating event analyzed per trace (103 traces for *At*MSL1, 34 for *At*MSL1 A320V). Source data are provided as a Source Data file.

Like TM3a in *Ec*MscS^24,36^, the pore-lining helix TM5a in *At*MSL1 contains several glycine and alanine residues with small side chains, which give rise to tight helix-helix interfaces in the closed conformation (Supplementary Fig. 3, Fig. 3a, b). Perturbation of this critical interface by the introduction of a bulkier side chain at position 106 in *Ec*MscS (A106V) resulted in an X-ray structure in a presumed open conformation^16^. This position, strategically located between two consecutive pore narrowing residues (L105 and L109), is highly conserved among MscS homologs and appears to be essential for the tight helix packing that stabilizes the closed conformation (Fig. 3a, b). Inspired by these observations, we introduced the equivalent mutation in *At*MSL1 (A320V) to evaluate whether analogous destabilization accordingly favors an open conformation.

Electrophysiological analyses of *Ec*MscS, *At*MSL1, and *At*MSL1 A320V expressed in giant *E. coli* spheroplasts indicated that *At*MSL1 A320V exhibits much longer open dwell times (that is, the amount of time spent in the open or partially open states) upon tension release than *At*MSL1 when gating is triggered by a brief application of negative pressure (Fig. 3c). Once gated, 90% of *At*MSL1 gating events had open dwell times of less than 20 s, compared to 44% of *At*MSL1 A320V gating events (Fig. 3d). Only 2% of *At*MSL1 gating events had open dwell times above 80 s, compared to 50% of *At*MSL1 A320V gating events.

Both *At*MSL1 and *At*MSL1 A320V exhibited subconducting states, but this was more frequently observed with *At*MSL1 A320V (Table 2, Fig. 3c). Furthermore, *At*MSL1 A320V was more likely than *At*MSL1 to open immediately after patch formation and prior to the application of pressure ramps as the patch of membrane within the glass pipette has inherent lateral tension due to the lipid-glass adhesion force (Table 2). *Ec*MscS, *At*MSL1, and *At*MSL1 A320V had statistically indistinguishable unitary conductances and relative gating pressure thresholds (0.49 ± 0.16, 0.64 ± 0.14, and 0.55 ± 0.15, respectively) when measured at a membrane potential of −70 mV (Table 3). However, unlike *Ec*MscS^14,26^, neither *At*MSL1 nor *At*MSL1 A320V displayed inactivation in response to repeated pressure ramps, regardless of ramp rate or length. Thus, the A320V mutation is likely to stabilize the open state of *At*MSL1 or to kinetically impede its closure.

**Table 2:** Spontaneous opening and subconducting states for *At*MSL1 and A320V.

	*At*MSL1	*At*MSL1 A320V
Channel(s) open before pressure ramp	25% (3/12 traces)	57% (8/14 traces)
Channel exhibits subconducting state	29% (30/102 traces)	50% (17/34 traces)
Subconducting state follows full opening	17% (5/30 traces)	82% (14/17 traces)

**Table 3:** *Ec*MscS and *At*MSL1 and A320V have similar conductances and relative gating pressures.

	*Ec*MscS	*At*MSL1	*At*MSL1 A320V
Conductance (nS)	1.02 ± 0.17	1.12 ± 0.16	0.98 ± 0.12
Relative gating pressure (*P*_*x*_/*P*_MscL_)	0.49 ± 0.16	0.63 ± 0.14	0.55 ± 0.15

Channel activities were triggered by application of 2.5 s symmetric pressure ramps at membrane potentials of −70 mV. Values are averages ± standard deviation. No difference in conductance or gating pressure relative to endogenous *Ec*MscL was observed between wild-type *At*MSL1 and A320V using one-way ANOVA followed by post hoc Tukey’s test, *p* < 0.05. *N* = 10 patches per variant. Source data are provided as a Source Data file.

**Fig. 4:**
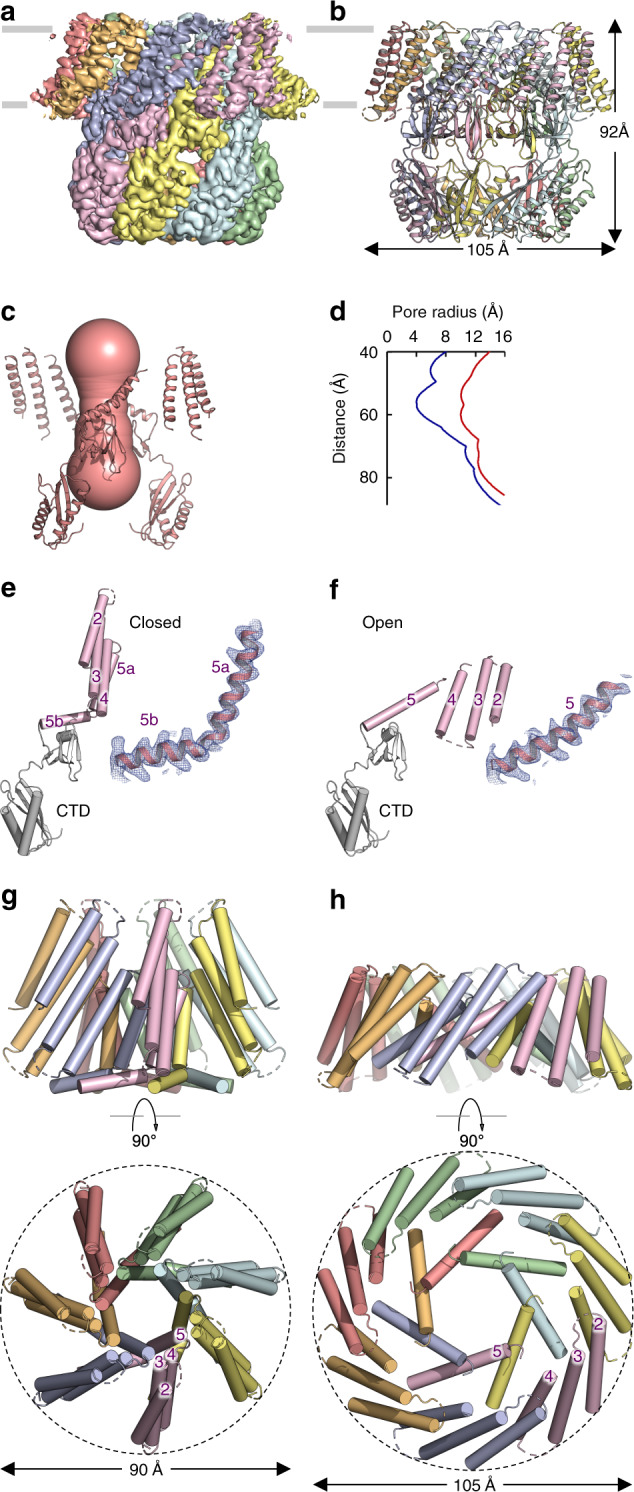
Structure of A320V and channel opening. **a** Cryo-EM density, contoured at 5.0 σ, for the A320V mutant. Each subunit is in a unique color. **b** Structure of A320V. The dimension of the channel is indicated. **c** The wide-open pore of A320V. **d** The pore profiles of the closed wild type (blue) and the open A320V mutant (red). **e**, **f** Structures of a single subunit for the wild type (**e**) and the A320V mutant channels (**f**). The C-terminal extramembrane domain in the mitochondrial matrix is colored in gray and the transmembrane region is colored in magenta. Cryo-EM densities, contoured at 7.0 σ, are shown for TM5. **g**, **h** Orthogonal views of the transmembrane domains of the wild-type MSL1 (**g**) and the A320V mutant (**h**).

We determined the cryo-EM structure of the *At*MSL1 A320V mutant in detergents to an overall resolution of ~3.0 Å (Fig. 4a, b, Supplementary Fig. 6, Table 1). Side views of the reference-free 2D projections indicate marked changes in the transmembrane region of A320V compared with that of the wild-type *At*MSL1 (Supplementary Fig. 7a–c). The bowl-shaped transmembrane region observed in the wild-type channel flattens, which is accompanied by considerable in-plane expansion. Flattening and expansion were recapitulated in reconstitution of A320V into lipid nanodiscs (Supplementary Fig. 7d), though A320V-nanodiscs did not yield high-resolution 3D reconstruction. In the structure of A320V in detergents, the diameter of the transmembrane domain is increased from 90 to 105 Å while the height of the channel is reduced from 116 to 92 Å, compared with those in the wild-type structure (Fig. 4a, b). These drastic global conformational changes in the transmembrane domain result in substantial opening of the central pore, which increases in diameter from ~8 Å in the wild type to ~20 Å in A320V (Fig. 4c, d). Therefore, we conclude that the A320V structure represents an open, conducting conformation.

### Gating mechanism

On the basis of these two structures, we can infer that the transition from the closed to the open state of MSL1 involves pronounced structural rearrangements in the transmembrane domain, whereas the soluble domains in the mitochondrial matrix remain essentially unchanged (Fig. 4e, f, Supplementary Movie 1) (r.m.s.d of ~1.1 Å for Cα atoms of residues 341–491). Upon channel opening, the peripheral helices TM2-4 approximately move as a rigid body by a rotation of ~135° (approximately about the helical axis of TM3) and a translation of ~30 Å. These conformational changes give rise to a flattened and expanded transmembrane domain in a lipid bilayer, in which transmembrane helices are more densely associated (Fig. 4g, h). The pore-lining helix TM5a becomes more tilted within the membrane and joins TM5b without an apparent kink (Fig. 4e, f). Concomitantly, the outward movement of the straightened TM5 helix results in wide opening of the hydrophobic gate (Fig. 4h, Supplementary Fig. 8), which renders the conduction path less hydrophobic at this region (Supplementary Fig. 9). The increased pore diameter and decreased hydrophobicity support our conclusion that the A320V structure represents an open, conductive conformation. Consistently, straightening of the pore-lining helix was suggested in a simulated open *Ec*MscS conformation, which was further supported by patch-clamp experiments of alanine substitutions at positions near the kink^24^. In the open structure of *At*MSL1 A320V, fewer lipid-attributable densities are observed between transmembrane helices than in the closed state (Supplementary Fig. 8), which is in accordance with the proposal that channel-bound lipids repartition to the bulk membrane bilayer under increased membrane tension to promote channel opening in *Ec*MscS^18^.

Thus, a single mutation in the pore-lining helix facilitates channel opening in both prokaryotic and eukaryotic MscS channels. However, conformational changes involved in channel gating show important similarities and differences between *Ec*MscS and *At*MSL1 (Supplementary Movies 1, 2; Supplementary Fig. 9). While the soluble domains remain virtually stationary during gating, the transmembrane domains experience substantial rearrangements. Both *Ec*MscS and *At*MSL1 channels appear to possess a peripheral ‘tension sensor’ (TM1-2 in *Ec*MscS and TM1-4 in *At*MSL1) that is attached to the pore-lining helix and undergoes dramatic repositioning upon activation. However, the direction and nature of the ‘tension sensor’ movements of the two channels are in marked contrast. Ultimately, the pore-lining helix becomes more perpendicular to the membrane plane in *Ec*MscS^16,18^, but more tilted in *At*MSL1 upon channel opening.

## Discussion

For a channel embedded in a membrane with lateral tension (*σ*), the free energy difference between the closed and open conformations can be expressed as

1 $$\Delta G = (\Delta G_{\mathrm{channel}} + \Delta G_{\mathrm{bending}}) - \sigma \Delta A,$$

where Δ*G*_channel_ is the difference of free energy intrinsic to channel gating, Δ*G*_*bending*_ the free energy difference of membrane bending associated with channel gating, and Δ*A* the difference of in-plane cross-sectional area of the channel^32,37^. Here, the closed and open structures of *At*MSL1 provide a straightforward structural mechanism for gating by lateral membrane tension. Under low tension, the transmembrane region of *At*MSL1 is curved. Increased lateral tension favors a more planar bilayer, which drives flattening of the transmembrane ‘tension sensor’, resulting in an in-plane area expansion of the transmembrane region (~23 nm^2^ for resolved TM2-TM5 helices) as well as straightening and outward movement of the pore-lining helices that ultimately open the hydrophobic gate. This simple but elegant concept of membrane flattening and area expansion has been previously suggested for mechanical gating of the architecturally unrelated Piezo1 channel on the basis of its intrinsically curved transmembrane region^32^. Indeed, force-induced flattening and expansion of Piezo1 have been indicated by atomic force microscopy studies^38^, but no open structure for Piezo channels has been determined. In this work we provide a near-atomic visualization of the membrane flattening and expansion process involved in channel gating. These results may be applicable to MscS homologs harboring even more transmembrane helices, and additional helices could function as an extension of the ‘tension sensor’ that undergoes force-induced flattening and expansion. Therefore, the structural transition illuminated in this study may represent a unified gating mechanism that underlies numerous mechanotransduction events in all kingdoms of life.

## Methods

### Cloning and expression and purification

DNA encoding *Arabidopsis thaliana* MSL1 (*At*MSL1, NCBI: NP_567165.2) was synthesized (Gene Universal Inc.) and served as the template for subsequent cloning. The N-terminal 79 amino acids corresponding to the mitochondrial targeting sequence was removed from the expression construct. Primers 5′-TAGCCTCGAGCCACCATGAGCAGCAAAAGCGATG-3′ and 5′-CCTTGAAACAAAACTTCCAAAGAATTCGA-3′ were used to generate the DNA fragment encoding residues 80–497 of *At*MSL1, which was ligated into a modified yeast *Pichia pastoris* expression vector pPICZ-B that contains a C-terminal PreScission protease cleavage site and a GFP-His_10_ tag. The A320V mutation was generated by site-directed mutagenesis using the primers 5′- GTTACCGCCTTTGCCGCCCGTG-3′ and 5′- GACGCCACCGACACCACCG-3′.

For expression in *E. coli* spheroplasts, *At*MSL1 residues 80–497, codon-optimized for expression in *E. coli*, were synthesized (ThermoFisher Scientific). This fragment was used to replace His-MSL1 in pET300-His-MSL1^28^ by Gibson cloning. The resulting pET300-*At*MSL1 vector was then linearized and a C-terminal GFP tag preceded by an EcoRI restriction site added by Gibson cloning to pET300-*At*MSL1 to create pET300-*At*MSL1-GFP. To create pET300-MscS-GFP, the *Ec*MscS sequence was used to replace the *At*MSL1 sequence in pET300-*At*MSL1-GFP using Gibson cloning. The A320V mutation was introduced into pET300-*At*MSL1-GFP by site-directed mutagenesis using the primers 5′- GTTGGTGGCGTTGTGACCGCATTTGC-3′ and 5′- GCAAATGCGGTCACAACGCCACCAAC-3′, creating pET300-A320V-GFP.

The wild-type *At*MSL1 channel and the A320V mutant were expressed in *Pichia pastoris* (strain SMD1163H, Invitrogen). Cells were harvested and disrupted by milling (Retsch MM400) and resuspended in buffer containing 50 mM Tris-HCl pH 8.0, 150 mM NaCl, a mixture of protease inhibitors (2.5 μg ml^−1^ leupeptin, 1 μg ml^−1^ pepstatin A, 100 μg ml^−1^ 4-(2-Aminoethyl) benzenesulfonyl fluoride hydrochloride, 3 μg ml^−1^ aprotinin, 1 mM benzamidine and 200 μM phenylmethane sulphonylfluoride) and DNase I. Cell membranes were solubilized by adding Lauryl Maltose Neopentyl Glycol (LMNG, Anatrace) to a final concentration of ~1% (w:v) while stirring for 2 h at 4 °C. Solubilized protein was separated from the insoluble fraction by centrifugation for 0.5 h at 30,000 × *g*. The suspension was incubated with 3 ml of cobalt-charged resin (G-Biosciences) for 3 h at 4 °C with rotation. Resin was then washed with 30 ml buffer containing 20 mM Tris-HCl pH 8.0, 150 mM NaCl, 20 mM imidazole, and 85 μM glyco-diosgenin (GDN, Anatrace). The GFP-His_10_ tag was removed by PreScission protease at 4 °C overnight with gentle rocking. The flow-through containing the channel protein was then collected, concentrated, and further purified on a Superose 6 increase 10/300 GL column (GE Healthcare Life Sciences) in 20 mM Tris-HCl pH 8.0, 150 mM NaCl and 40 μM GDN. The peak fractions were combined and concentrated for cryo-EM experiments.

### Nanodisc reconstitution

Soybean polar lipid extract (Avanti Polar Lipids, Inc.) in chloroform was dried to form a thin film in a glass tube under argon and then by vacuum desiccation for over 2 h. The lipid film was rehydrated to 10 mM in buffer containing 20 mM Tris-HCl pH 8.0, 150 mM NaCl, and 14 mM DDM, and sonicated immediately before use. Protein with C-terminal GFP-His_10_ tag was eluted from the cobalt-charged resin and concentrated to ~45 μM and then mixed with the scaffold protein MSP2N2 and lipids in a final molar ratio of ~1:0.5:50. The mixture was incubated on ice for 10 min before the addition of Bio-beads SM-2 (Bio-Rad) to a final volume of ~12.5% (v/v) to remove the detergent. In the meanwhile, PreScission protease was also added to cleave the C-terminal GFP-His_10_ tag. The resulting mixture was incubated at 4 °C overnight with constant rotation. The supernatant was isolated by centrifugation, concentrated and further purified on a Superose 6 increase 10/300 GL column (GE Healthcare Life Sciences) in buffer containing 20 mM Tris-HCl pH 8.0 and 150 mM NaCl.

### Cryo-EM sample preparation and imaging

Cryo-EM grids were prepared with FEI Vitrobot Mark IV (FEI). 3.5 μl of purified channel protein (~6 mg ml^−1^ in detergent micelles or ~3 mg ml^−1^ in lipid nanodiscs) was applied onto glow-discharged copper Quantifoil R2/2 holey carbon grids (Quantifoil). Grids were blotted for 2 s at ~100% humidity and flash frozen in liquid ethane. For imaging, the grids were loaded into a Titan Krios (FEI) electron microscope operating at 300 kV, which is equipped with GIF Quantum energy filter and a Gatan K2 Summit (Gatan) detector. Movies were recorded using the EPU software (https://www.fei.com/software/epu-automated-single-particles-software-for-life-sciences/) in super-resolution mode with a pixel size of 0.55 Å and a nominal defocus value between −1.0 and −2.5 μm. Data were collected with a dose of ~7.8 electrons per Å^2^ per second, and each movie was recorded by 40 frames (200 ms per frame) for an 8 s exposure.

### Image processing and map calculation

Image stacks were first aligned, binned by 2, and dose-weighted using MotionCor2^39^, and then subjected to contrast transfer function (CTF) determination using GCTF^40^. Following motion correction and CTF estimation, low-quality images were manually removed from the datasets. For *At*MSL1 in detergent GDN, 1031 particles were manually selected to compute two-dimensional class templates for automated particle picking in RELION3^41^. A total of 936,130 particles were automatically picked from 3618 micrographs. A box size of 256 pixels was used for particle extraction and a mask diameter of 190 Å was used for 2D classification. Two rounds of 2D classification were performed to identify particles representing the channel (454,555 particles), which were imported into cryoSPARC^42^ to generate an initial map for 3D classification in RELION3. Three classes showing intact channel features (158,172 particles) were selected and subjected to 3D refinement, yielding an overall resolution of 3.48 Å. CTF refinement and Bayesian polishing further improved the resolution to 3.06 Å after masked 3D refinement.

For *At*MSL1 in nanodiscs, 2595 particles were manually picked to generate 2D class templates for automated particle picking in RELION3, which resulted in a total of 457,683 particles from 3486 micrographs. After two rounds of 2D classification, 140,548 particles were selected for 3D classification using low-pass filtered map of *At*MSL1 in detergent micelles as the initial model. One class (23,077 particles) was selected for 3D refinement, reaching an overall resolution of 4.2 Å. CTF refinement and Bayesian polishing improved the resolution to 3.39 Å after masked 3D refinement.

For the *At*MSL1 A320V mutant in detergents, 5553 particles were picked using LoG-based auto-picking to generate 2D classes for automatic picking in RELION3. Automatic picking resulted in 428,889 particles from 1947 micrographs. From two rounds of 2D classification, 192,899 particles were selected and imported into cryoSPARC to calculate an initial map for 3D classification requesting four classes in RELION3. Two classes (96,781 particles) containing clear TM densities were selected for further 3D refinement, reaching an overall resolution of 3.86 Å. 3D classification without image alignment was performed and two classes (74,578 particles) were selected for further 3D refinement, yielding a 3.81 Å reconstruction. CTF refinement and Bayesian polishing improved the resolution to 3.27 Å after 3D refinement. 3D classification without image alignment using the polished particles was performed, and the major class containing 52,883 particles was selected for masked 3D refinement, reaching a final overall resolution to 2.96 Å.

For A320V in nanodiscs, 2016 particles were manually selected for 2D class templates. Automatic picking resulted in 464,730 particles from 3234 micrographs. From two rounds of 2D classification, 65,310 particles were selected. However, 3D reconstruction did not reach resolution beyond 8 Å in cryoSPARC and RELION3, and therefore we did not build the model.

### Model building and coordinate refinement

A homology model of the *At*MSL1 extramembrane domain was generated using coordinates of *Ec*MscS (PDB: 5AJI) by the SWISS-MODEL server^43^. The model was placed into the cryo-EM density map by using UCSF Chimera^44^ and then manually rebuilt in COOT^45^. The transmembrane region was de novo built in COOT. Iterative model building in COOT and refinement using real_space_refine in PHENIX^46^ were performed to obtain the final model. The final model shows good geometry and contains amino acids 203–229, 236–269, and 276–491. The structure of *At*MSL1 in lipid nanodiscs was built using the *At*MSL1 structure in detergent micelles as a reference, followed by multiple rounds of model rebuilding in COOT and refinement using real_space_refine in PHENIX. For the *At*MSL1 A320V mutant, resolution of the EM density map is higher in the C-terminal extramembrane domain. Density for the transmembrane region is weaker. The C-terminal domain of the wild-type *At*MSL1 was first placed into the EM density map as a rigid body. Owing to weaker density in the transmembrane domain, TM5 was de novo built in COOT, and this partial model comprising the C-terminal domain and TM5 was refined against the map using real_space_refine in PHENIX. In the final steps, TM2-TM4 helices from the wild-type channel were modeled into the A320V cryo-EM density map as a rigid body, and then further refined. The final A320V structure contains residues 204–229, 236–269, 276–302 and 313–491. Final refined models were validated using MolProbity^47^. Pore dimensions were estimated by using HOLE^48^. Structural figures, including the surface hydrophobicity plots^49^, and morphed movies (using the linear interpolation method) were prepared using PyMol (pymol.org) and UCSF Chimera^44^. The phylogenetic tree was generated by MEGA X^50^.

### Electrophysiology

*At*MSL1, A320V, and *Ec*MscS were expressed in giant spheroplasts made using *E. coli* strain MJF516, which lacks *mscS*, *mscK*, *ybiO*, and *yjeP*^51^. A lysogenized version of *E. coli* strain MJF516 (generated using the Novagen λDE3 lysogenization kit (Millipore Sigma)) was transformed with pET300-*Ec*MscS-GFP, pET300-*At*MSL1-GFP, or pET300-A320V-GFP. Giant spheroplasts were prepared following the protocol^52^ with the following modifications: treatment with cephalexin only was performed for 1 h, at which point isopropyl β-d-1-thiogalactopyranoside (IPTG) was added to a final concentration of 1 mM and cultures shaken at 42 °C, 180 rpm for an additional hour before being stored overnight at 4 °C in 50 mL conical tubes.

Patch-clamp electrophysiology was performed with inside-out excised patches. Pipette buffer contained 200 mM KCl, 90 mM MgCl_2_, 5 mM CaCl_2_, 5 mM Hepes, pH 7.4 and the bath buffer was identical to the pipette buffer with the addition of 400 mM sucrose. Pressure was controlled and applied using an HSPC-1 high speed pressure clamp system (ALA Scientific) and data acquired using an Axon Axopatch 200B amplifier and a Digidata 1440A digitizer (Molecular Devices). All pipettes were of bubble number ~4.5. Data were acquired at 20 kHz for pressure sensitivity and conductance measurements and at 10 kHz for open-state lifetime measurements and all data low-pass filtered at 5 kHz. Pressure sensitivity was evaluated at −70 mV membrane potential using a symmetric pressure ramp consisting of a 2.5 s increase in applied suction followed by a 2.5 s suction release. Open state lifetime measurements were performed at a membrane potential of −70 mV and consisted of a symmetric pressure ramp of 1 s suction increase followed by 1 s suction release and continued application of 70 mV pipette potential without additional suction for 95.7 s after suction release.

Data analysis and measurements were performed using ClampFit 10.6 software (Molecular Devices). For all measurements, traces in which channel gating was observed prior to pressure ramp application were discarded to ensure that only initial channel gating events were measured. The relative gating pressures of each channel, *P*_*X*_/*P*_MscL_, where *X* is either *At*MSL1, A320V, or *Ec*MscS was calculated using the gating pressure at which the first sustained channel opening occurred^36^. For relative gating pressure and conductance calculations, values from all possible traces for a given patch were averaged, then the average and standard deviations of all patch averages calculated. Patch averages of conductance and relative gating pressures were analyzed using one-way ANOVA followed by post hoc Tukey’s tests to test for significant differences in relative gating pressure and conductance between *At*MSL1, A320V, and *Ec*MscS.

To determine open-state lifetimes of *At*MSL1 and A320V, the open dwell times of pressure-triggered channel openings were measured, with channels considered fully closed only when activity was lost for a period of 5 s or longer. Typically, only a single channel was observed in each patch; if multiple channels opened, only the first was used for analysis. Open dwell times observed for each channel were then binned into the following categories: 0–19.99 s, 20–39.99 s, 40–59.99 s, 60–79.99 s, 80+ s, and the percentage of openings in each category calculated for *At*MSL1 and A320V. Pipette potential was unclamped at 97.7 s.

### Reporting summary

Further information on research design is available in the Nature Research Reporting Summary linked to this article.

## Supplementary information

SUPPLEMENTARY INFO

Peer Review File

Supplementary Movie 1

Supplementary Movie 2

Reporting Summary

## Data Availability

Data supporting the findings of this manuscript are available from the corresponding author upon reasonable request. A reporting summary for this Article is available as a Supplementary Information file. Source data are provided with this paper. The cryo-EM maps have been deposited to Electron Microscopy Data Bank with accession codes EMD-21444, EMD-21445, and EMD-21447. Atomic coordinates have been deposited to the Protein Data Bank (PDB) with accession codes: PDB 6VXM, PDB 6VXN, PDB 6VXP.

## Source data

Source Data
