# Supplementary material for: Structural mechanism for gating of a eukaryotic mechanosensitive channel of small conductance
Source: Nat Commun. 2020 Jul 23;11:3690. doi: 10.1038/s41467-020-17538-1 (PMC7378837; doi:10.1038/s41467-020-17538-1)
Supplement: Supplementary file 1 — SUPPLEMENTARY INFO [file 41467_2020_17538_MOESM1_ESM.pdf]

Supplementary Information

Structural mechanism for gating of a eukaryotic mechanosensitive channel of small conductance

Deng et al.

9 Supplementary Figures

2 Supplementary Movies

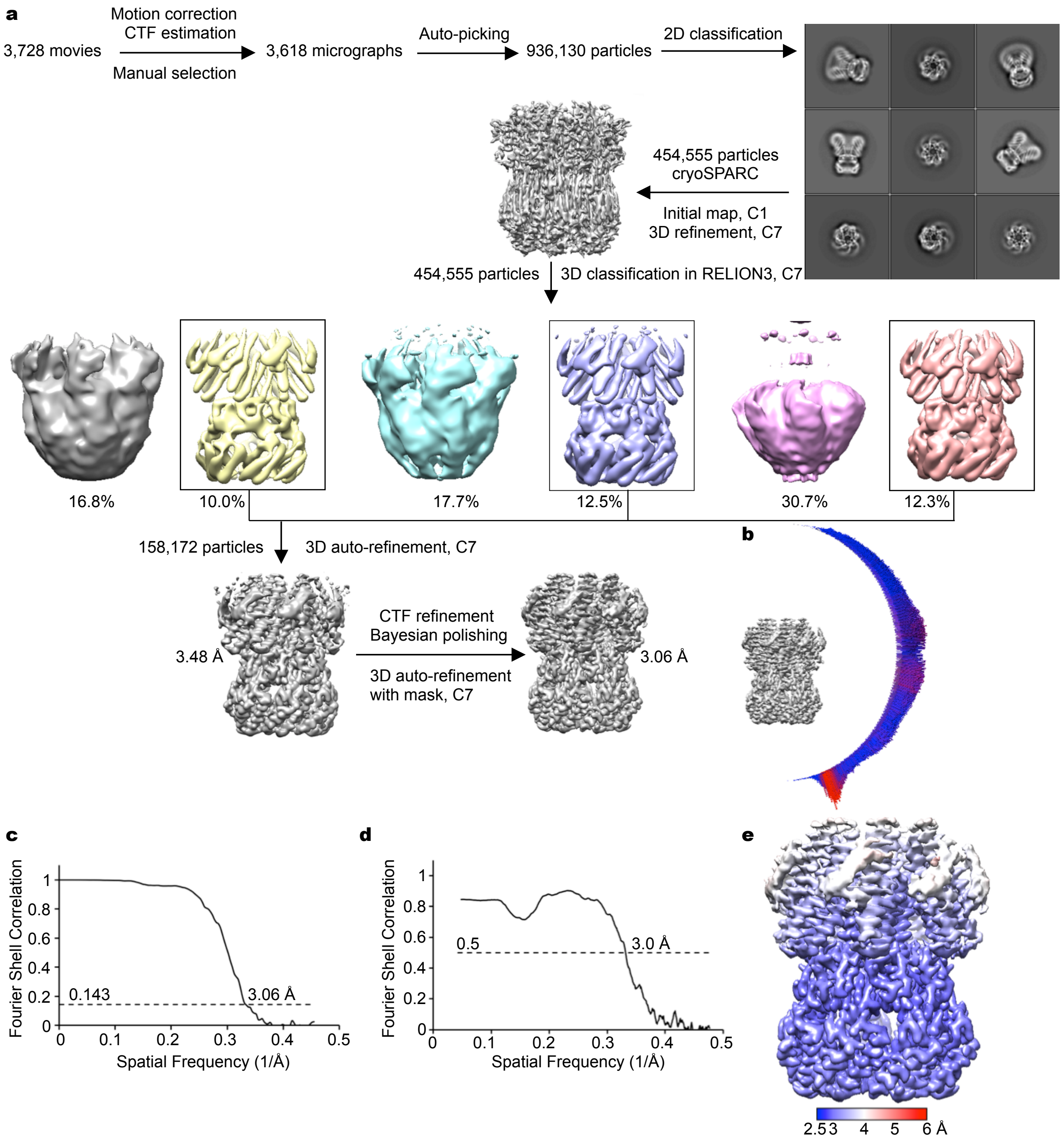

Supplementary Fig. 1 Cryo-EM reconstruction of *At*MSL1 in detergents. a, Schematic of cryo-EM data processing. **b**, Orientation distribution of particles used for final reconstruction. **c**, Fourier shell correlation of the final map. **d**, Fourier shell correlation between the refined model and the full map. **e**, Cryo-EM density map colored by local resolution.

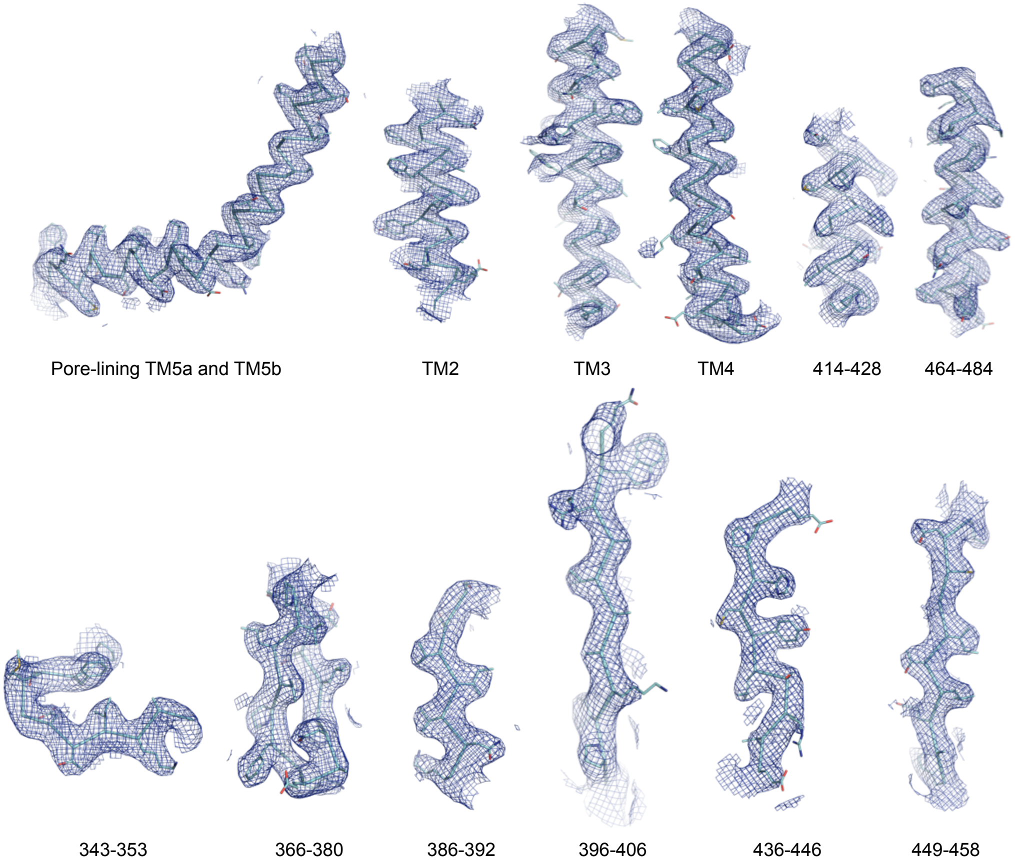

Supplementary Fig. 2 Representative cryo-EM density of *At*MSL1 in detergents.

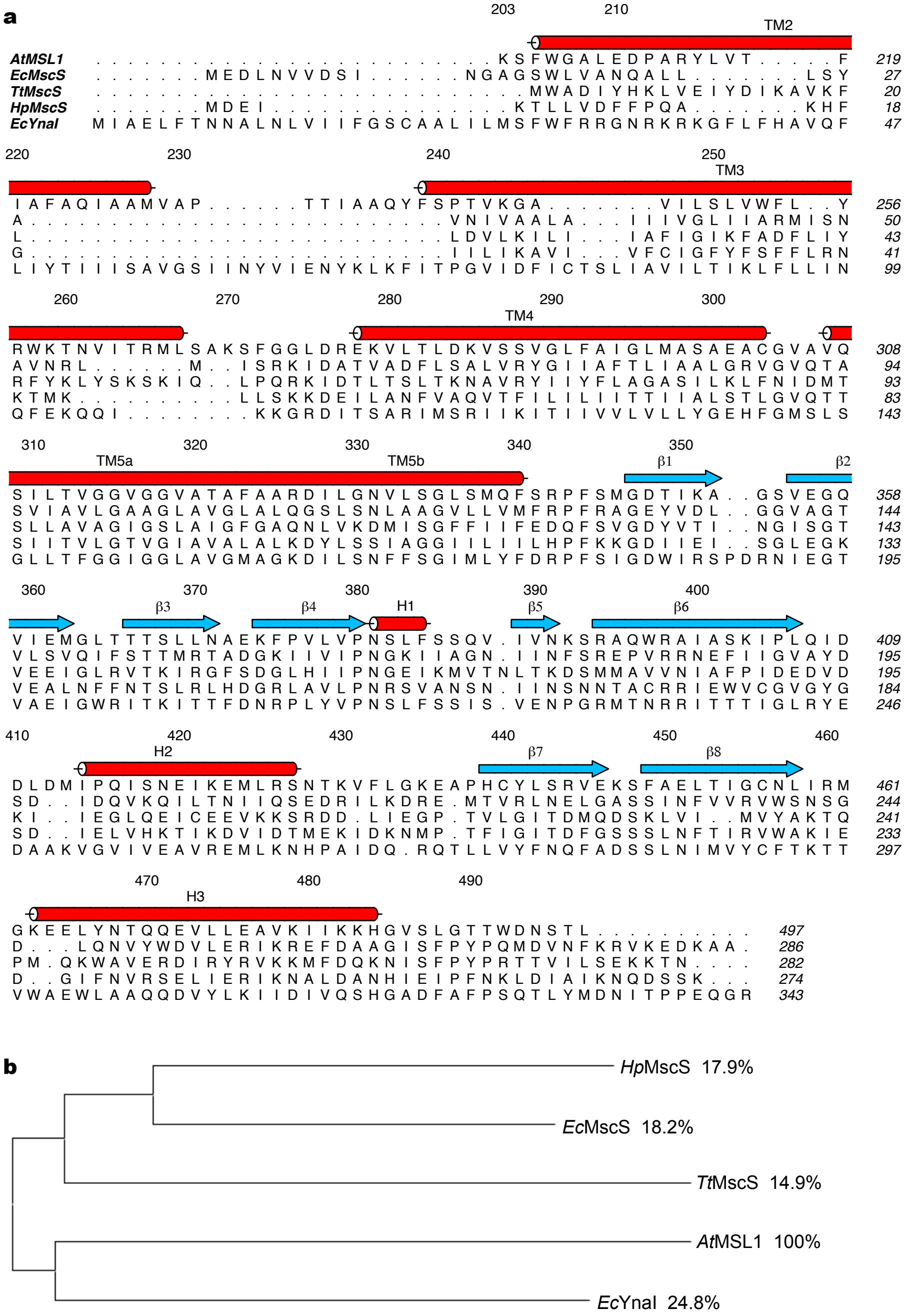

Supplementary Fig. 3 Sequence comparison of MscS homologs. a, Sequence alignment of MscS homologs with known structures. The protein sequences of *Arabidopsis thaliana* (*At*MSL1, NCBI sequence: NP_567165.2), *E. coli* MscS (*Ec*MscS, NCBI sequence: NP_417399.1), *Thermoanaerobacter tengcongensis* (*Tt*MscS, NCBI sequence: AAM25887.1), and *Helicobacter pylori* MscS (*Hp*MscS, NCBI sequence: WP_021304837.1), and *E. coli* YanI (*Ec*YnaI, NCBI sequence: VWQ02384.1) are aligned and secondary structure elements of *At*MSL1 are shown above the protein sequences. **b**, Phylogenetic tree of MscS homologs with sequence identity to *At*MSL1.

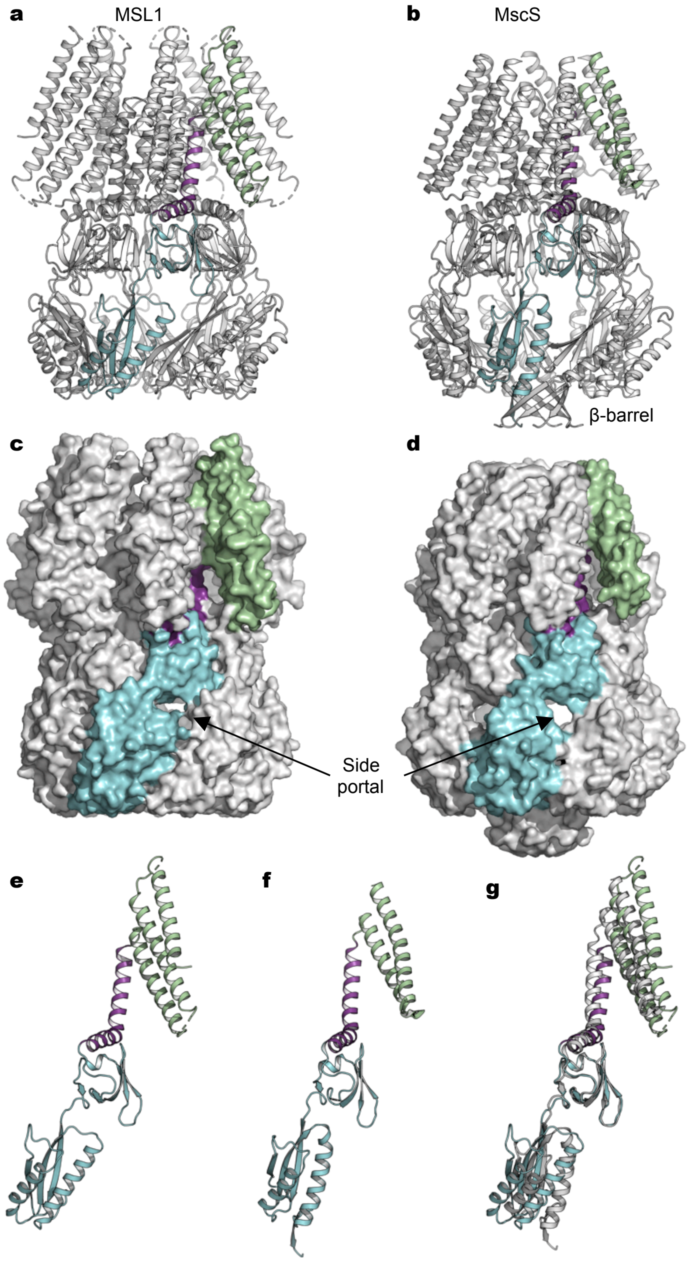

Supplementary Fig. 4 Structural comparison of *At*MSL1 and *Ec*MscS. a,**b**, Structures of *At*MSL1 (**a**) and *Ec*MscS (PDB: 2OAU) (**b**). One of the subunits is uniquely colored. The C-terminal β-barrel observed in *Ec*MscS is absent in *At*MSL1. **c**,**d**, Surface representations of *At*MSL1 (**c**) and *Ec*MscS (**d**). The side portals are indicated. **e**,**f**, Structures of a single channel subunit of *At*MSL1 (**e**) and *Ec*MscS (**f**). **g**, Overlay of single subunits of *At*MSL1 in colors and *Ec*MscS in gray. The structures were aligned using the most conserved regions (amino acids 327-386 in *At*MSL1 and 113-172 in *Ec*MscS, r.m.s.d of Cα atoms ~1.0 Å).

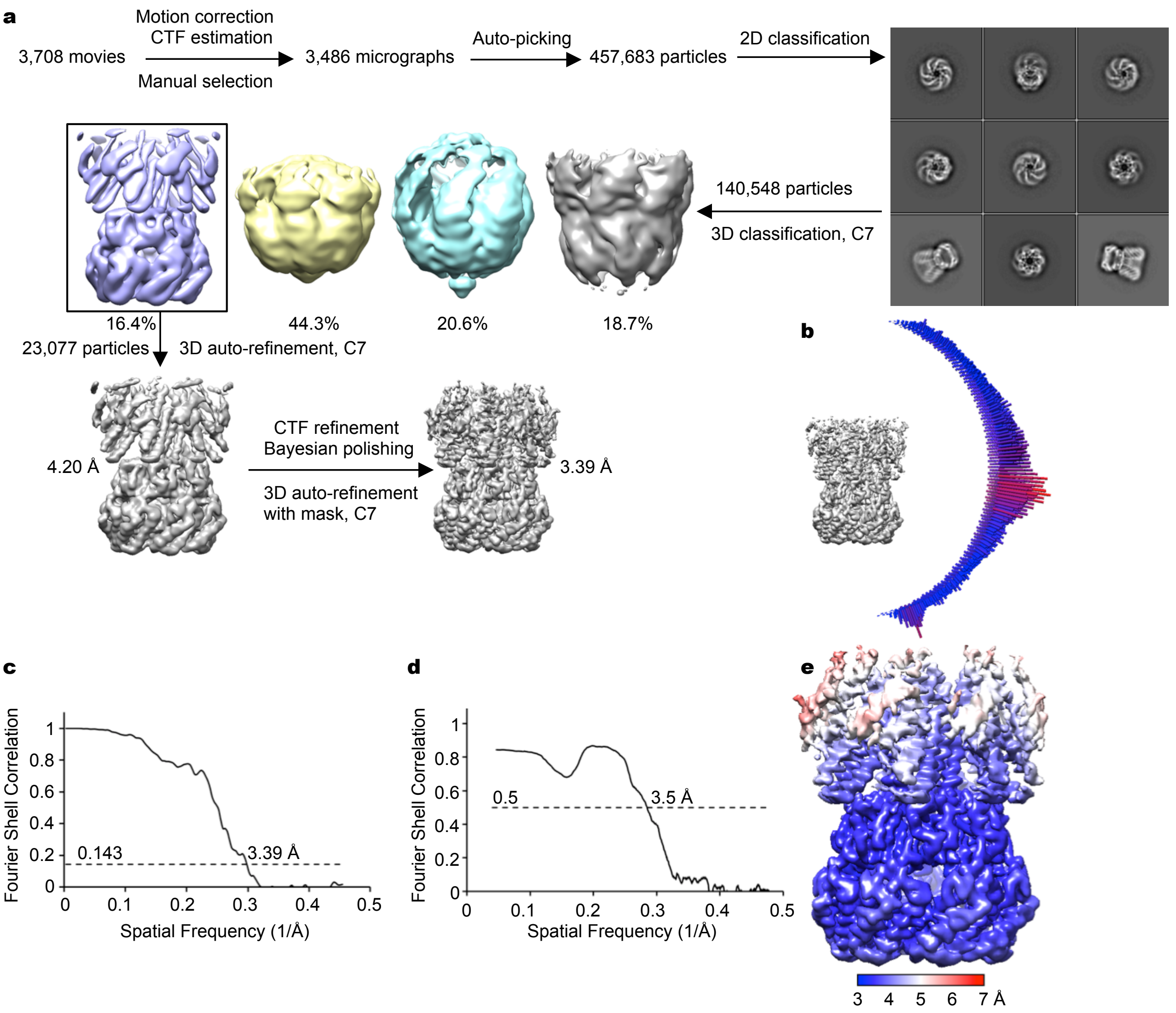

Supplementary Fig. 5 Cryo-EM reconstruction of *At*MSL1 in lipid nanodiscs. a, Schematic of cryo-EM data processing. **b**, Orientation distribution of particles used for final reconstruction. **c**, Fourier shell correlation of the final map. **d**, Fourier shell correlation between the refined model and the full map. **e**, Cryo-EM density map colored by local resolution.

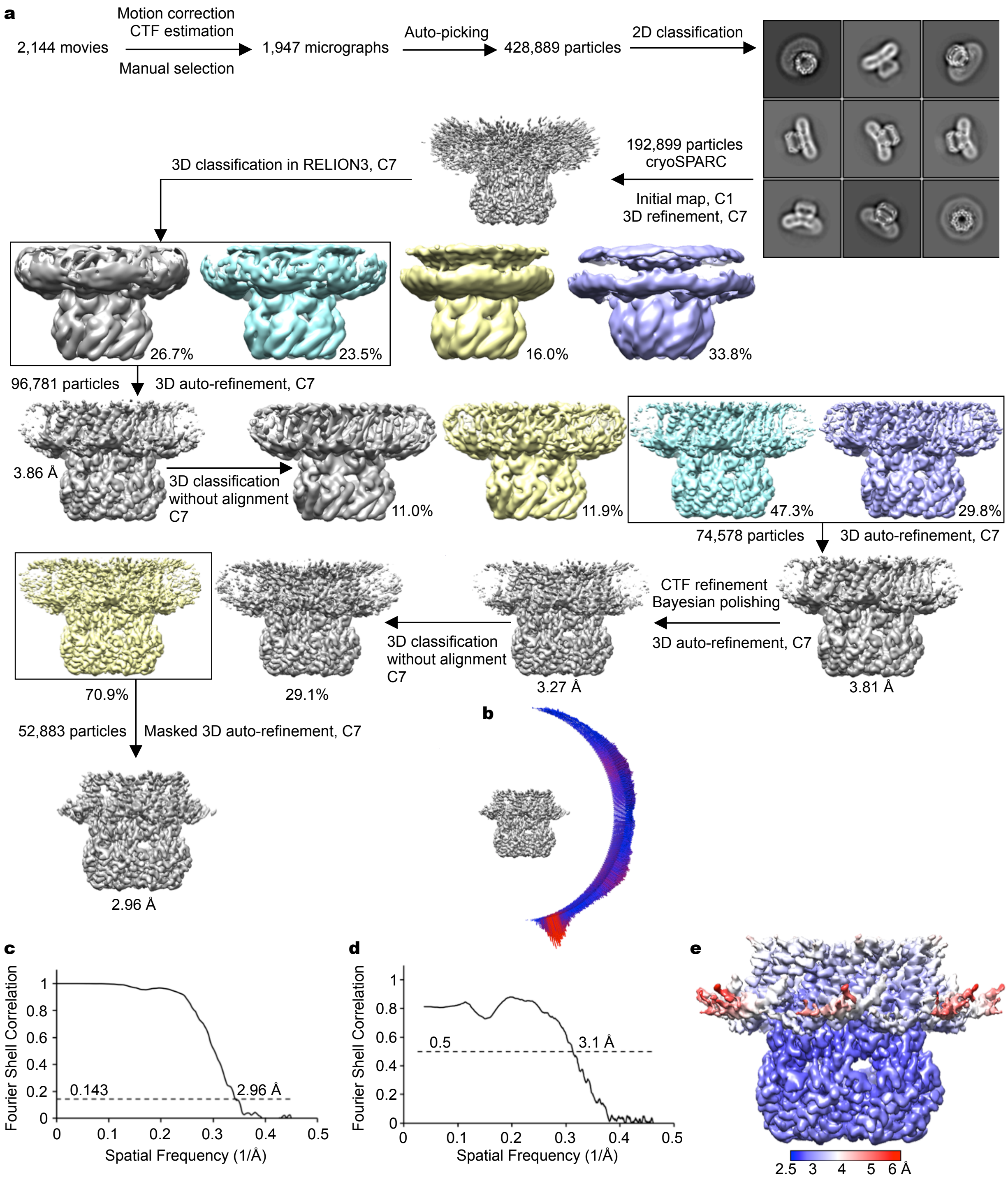

Supplementary Fig. 6 Cryo-EM reconstruction of the *At*MSL1 A320V mutant. a, Schematic of cryo-EM data processing. **b**, Orientation distribution of particles used for final reconstruction. **c**, Fourier shell correlation of the final map. **d**, Fourier shell correlation between the refined model and the full map. **e**, Cryo-EM density map colored by local resolution.

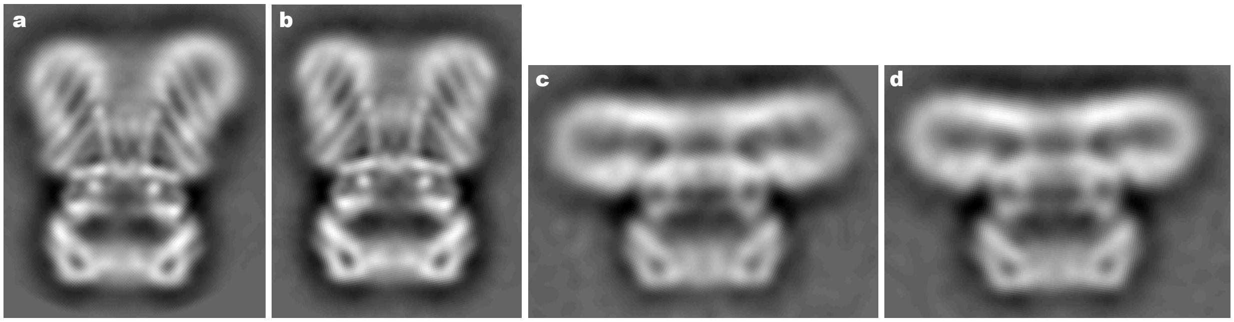

Supplementary Fig. 7 2D class averages for *At*MSL1 and A320V in detergents and in nanodiscs. a-d, 2D class averages representing side views of the wild-type *At*MSL1 in detergent micelles (**a**) and in lipid nanodiscs (**b**), and the *At*MSL1 A320V mutant in detergent micelles (**c**) and in lipid nanodiscs (**d**). The wild-type *At*MSL1 shows a bowl-like transmembrane region both in detergents and in nanodiscs, whereas A320V displays a flattened and expanded transmembrane region both in detergents and in nanodiscs. Identical detergent and nanodisc conditions are used for the wild type channel and the A320V mutant.

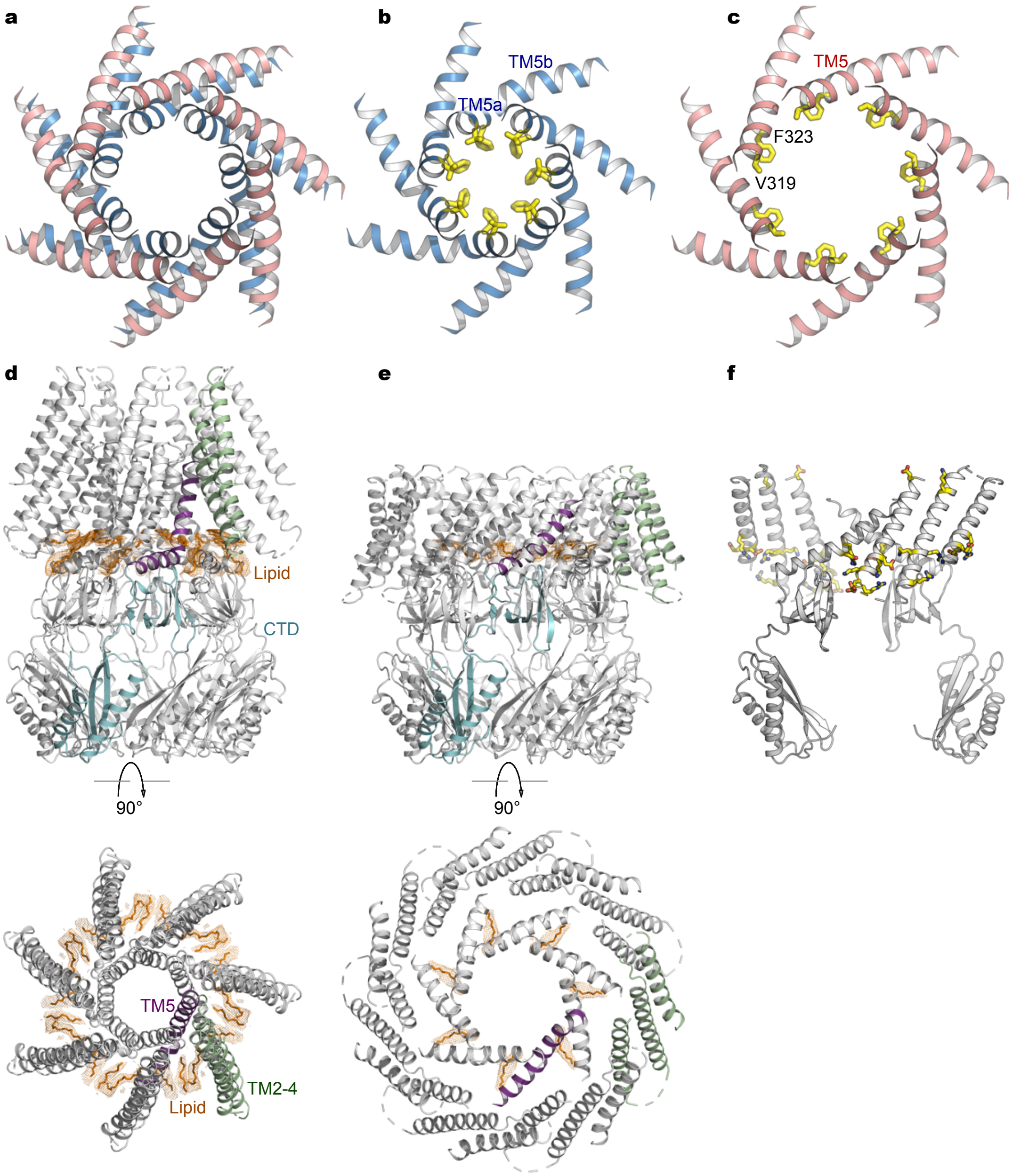

Supplementary Fig. 8 Conformational changes upon channel opening in *At*MSL1. a, Superposition of the closed (blue) and open (red) pores. **b**, The closed pore of *At*MSL1. **c**, The open pore of the *At*MSL1 A320V mutant. Hydrophobic gate residues (V319 and F323) are indicated with side chains shown in stick representation. **d**, Orthogonal views of the closed conformation of *At*MSL1. **e**, Orthogonal views of the open conformation of A320V. One of the channel subunits is uniquely colored. Lipid density contoured at 5.0 σ is shown in orange mesh. Only the transmembrane region is shown in the bottom panels. **f**, Distribution of charged amino acids in the transmembrane helices in the open structure.

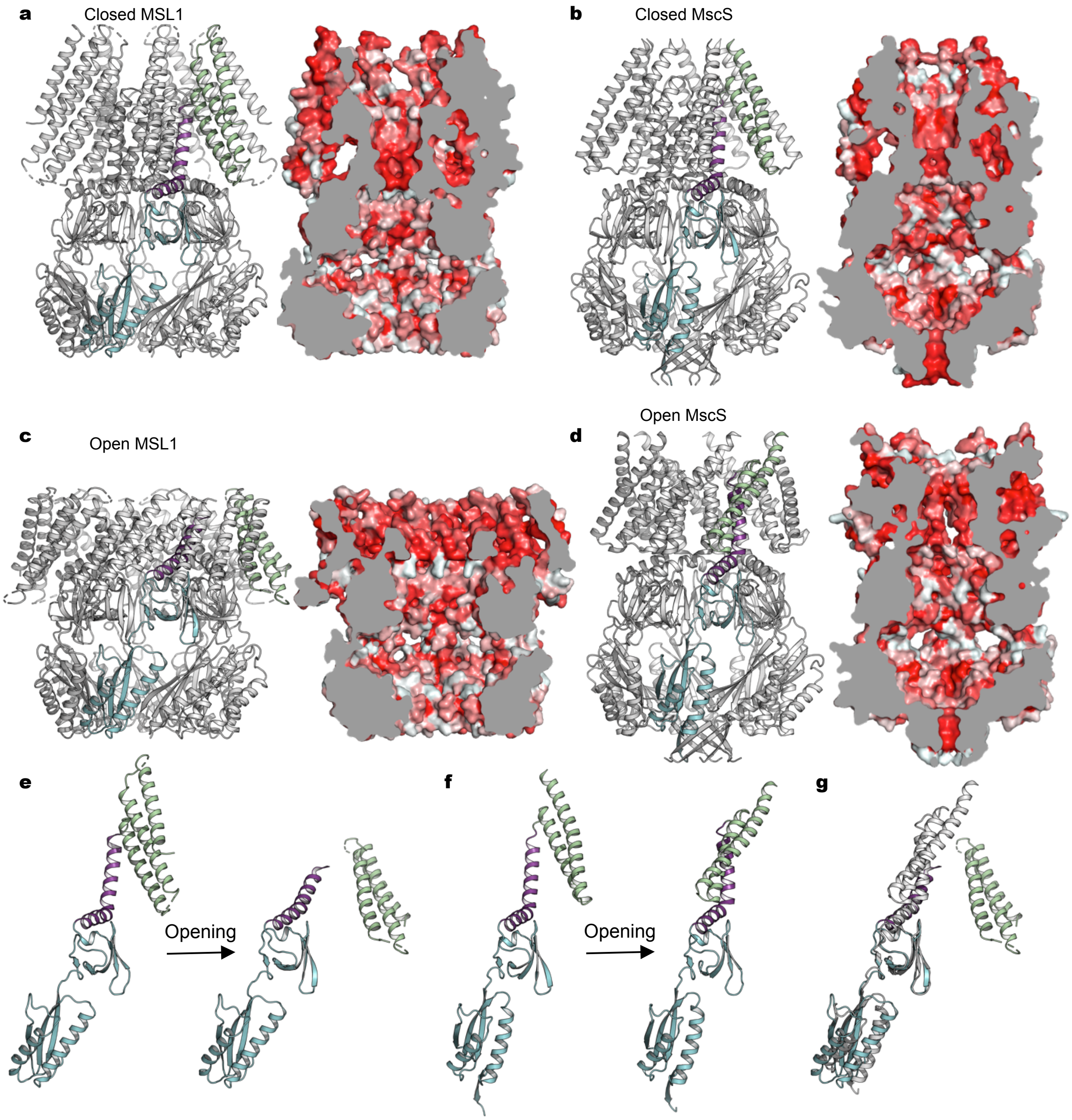

Supplementary Fig. 9 Opening of *At*MSL1 and *Ec*MscS. a,**b**, Presumably closed structures of *At*MSL1 (**a**) and *Ec*MscS (PDB: 6RLD) (**b**). One of the subunits is uniquely colored. **c**,**d**, Presumably open structures of *At*MSL1 (**c**) and *Ec*MscS (PDB: 2VV5) (**d**). The surface hydrophobicity is shown alongside each structure. Red and white indicate high and low hydrophobicity, respectively. **e**,**f**, Structural changes of a single channel subunit of *At*MSL1 (**e**) and *Ec*MscS (**f**) upon channel opening. **g**, Overlay of single subunits of open *At*MSL1 in colors and open *Ec*MscS in gray. The structures were aligned using the most conserved regions (amino acids 327-386 in *At*MSL1 and 113-172 in *Ec*MscS, r.m.s.d of Cα atoms ~1.1 Å).
