# Supplementary material for: Structural mechanism for gating of a eukaryotic mechanosensitive channel of small conductance
Source: Nat Commun. 2020 Jul 23;11:3690. doi: 10.1038/s41467-020-17538-1 (PMC7378837; doi:10.1038/s41467-020-17538-1)
Supplement: Supplementary file 5 — Reporting Summary [file 41467_2020_17538_MOESM5_ESM.pdf]

nature research

Corresponding author(s): Peng Yuan

Last updated by author(s): Jun 25, 2020

Reporting Summary

Nature Research wishes to improve the reproducibility of the work that we publish. This form provides structure for consistency and transparency in reporting. For further information on Nature Research policies, see Authors & Referees and the Editorial Policy Checklist.

Statistics

For all statistical analyses, confirm that the following items are present in the figure legend, table legend, main text, or Methods section.

n/a	Confirmed	
☐	☒	The exact sample size (*n*) for each experimental group/condition, given as a discrete number and unit of measurement
☐	☒	A statement on whether measurements were taken from distinct samples or whether the same sample was measured repeatedly
☐	☒	The statistical test(s) used AND whether they are one- or two-sided<br>*Only common tests should be described solely by name; describe more complex techniques in the Methods section.*
☒	☐	A description of all covariates tested
☒	☐	A description of any assumptions or corrections, such as tests of normality and adjustment for multiple comparisons
☐	☒	A full description of the statistical parameters including central tendency (e.g. means) or other basic estimates (e.g. regression coefficient) AND variation (e.g. standard deviation) or associated estimates of uncertainty (e.g. confidence intervals)
☐	☒	For null hypothesis testing, the test statistic (e.g. *F*, *t*, *r*) with confidence intervals, effect sizes, degrees of freedom and *P* value noted<br>*Give P values as exact values whenever suitable.*
☒	☐	For Bayesian analysis, information on the choice of priors and Markov chain Monte Carlo settings
☒	☐	For hierarchical and complex designs, identification of the appropriate level for tests and full reporting of outcomes
☒	☐	Estimates of effect sizes (e.g. Cohen's *d*, Pearson's *r*), indicating how they were calculated

Our web collection on statistics for biologists contains articles on many of the points above.

Software and code

Policy information about availability of computer code

Data collection	Cryo-EM: EPU version 1.11.1.5REL; Electrophysiology: pClamp 10.6 suite
Data analysis	Cryo-EM: MotionCor2 1.2.1, GCTF v1.06, RELION 3.0, cryoSPARC 2, UCSF CHIMERA 1.11.2 , COOT v0.8.9.2, PyMOL 2.3.2, HOLE, PHENIX 1.15.2-3472, MolProbity 4.4; Electrophysiology: pClam 10.6 suite; General data analysis: Microsoft Excel

For manuscripts utilizing custom algorithms or software that are central to the research but not yet described in published literature, software must be made available to editors/reviewers. We strongly encourage code deposition in a community repository (e.g. GitHub). See the Nature Research guidelines for submitting code & software for further information.

Data

Policy information about availability of data

All manuscripts must include a data availability statement. This statement should provide the following information, where applicable:

- Accession codes, unique identifiers, or web links for publicly available datasets
- A list of figures that have associated raw data
- A description of any restrictions on data availability

The cryo- EM maps have been deposited to Electron Microscopy Data Bank with accession codes EMD-21444, EMD-21445, and EMD-21447. Atomic coordinates have been deposited to the Protein Data Bank (PDB) with accession codes 6VXM, 6VXN, and 6VXP. Other source data are available from the corresponding authors upon request.

October 2018

Field-specific reporting

Please select the one below that is the best fit for your research. If you are not sure, read the appropriate sections before making your selection.

☒ Life sciences ☐ Behavioural & social sciences ☐ Ecological, evolutionary & environmental sciences

For a reference copy of the document with all sections, see nature.com/documents/nr-reporting-summary-flat.pdf

Life sciences study design

All studies must disclose on these points even when the disclosure is negative.

Sample size	Electrophysiological recordings were repeated at least five times. The sample size is determined based on data consistency.
Data exclusions	For cryo-EM reconstruction of the AtMSL1 in detergent, 936,130 particles were included in 2D and 3D classification. 777,958 particles resulting in low resolution in 2D classification or in 3D classification were discarded.<br>For cryo-EM reconstruction of the AtMSL1 in nanodiscs, 457,683 particles were included in 2D and 3D classification. 434,606 particles resulting in low resolution in 2D classification or in 3D classification were discarded.<br>For cryo-EM reconstruction of the AtMSL1 A320V in detergent, 428,889 particles were included in 2D and 3D classification. 376,006 particles resulting in low resolution in 2D classification or in 3D classification were discarded.
Replication	All experiments were reliably reproduced.
Randomization	For electrophysiology, cells expressing channels (indicated by GFP fluorescence) were randomly selected.
Blinding	The investigators were blinded to group allocation during data collection and analysis.

Reporting for specific materials, systems and methods

We require information from authors about some types of materials, experimental systems and methods used in many studies. Here, indicate whether each material, system or method listed is relevant to your study. If you are not sure if a list item applies to your research, read the appropriate section before selecting a response.

Materials & experimental systems

n/a	Involved in the study	
☒	☐	Antibodies
☐	☒	Eukaryotic cell lines
☒	☐	Palaeontology
☒	☐	Animals and other organisms
☒	☐	Human research participants
☒	☐	Clinical data

Methods

n/a	Involved in the study	
☒	☐	ChIP-seq
☒	☐	Flow cytometry
☒	☐	MRI-based neuroimaging

Eukaryotic cell lines

Policy information about cell lines

Cell line source(s)	Pichia pastoris
Authentication	Cell lines were not authenticated.
Mycoplasma contamination	Cell lines were not tested for Mycoplasma contamination.
Commonly misidentified lines (See ICLAC register)	*Name any commonly misidentified cell lines used in the study and provide a rationale for their use.*
