# Supplementary material for: Structural mechanism for gating of a eukaryotic mechanosensitive channel of small conductance
Source: Nat Commun. 2020 Jul 23;11:3690. doi: 10.1038/s41467-020-17538-1 (PMC7378837; doi:10.1038/s41467-020-17538-1)
Supplement: Supplementary file 2 — Peer Review File [file 41467_2020_17538_MOESM2_ESM.docx]

Peer Review Information

**Journal:** Nature Communications

**Manuscript title:** Structural mechanism for gating of a eukaryotic mechanosensitive channel of small conductance

**Corresponding author name(s):** Dr Peng Yuan

**Editorial notes:**

**none**

**Reviewer comments & decisions:**

**Reviewer comments, first version:**

Reviewer #1 (Remarks to the Author):

The paper ‘Structural mechanism for gating of a eukaryotic mechanosensitive channel of small conductance’ by Deng and coauthors is a substantial contribution to the field. MSL1 is the first 5-TM representative of the MscS family to be structurally characterized to a resolution that allows for an atomic model to be built for many critical parts of the protein. The important features of the revealed open-state conformation are: (1) the pore-lining helices are tilted and straight, such that the width of the open pore is now sufficient to account for the experimental conductance; and (2) there is a tight contact between the lipid-facing helices and the pore-lining TM5s that furnishes a force-transmission route from the lipid bilayer to the gate. These features were discussed in the field in the form of models, but now for the first time, they are explicitly seen in an open channel structure.
The paper reads well and the graphical material is clear and adequate.

The following information needs to be included.

1. Because EcMscS inactivates (refs 14, 26), and the initial crystal structure (2OAU) was proposed to represent this functional state (ref 24), the authors should say whether or not inactivation was observed in MSL1 under any experimental circumstances (tension, voltage, crowders, etc). This is not a random question because MSL1 fulfills its function under high voltage (150-180 mV) in the inner membrane of an energized mitochondrion. A single MSL1 opening will completely uncouple the energetics and for this reason, a ‘safe’ tension-insensitive and impermeable state might exist. If indeed MSL1 shows the ability to inactivate, it should be discussed if the state with uncoupled TM4 and TM5 helices is closed or inactivated.

2. The authors perform their patch-clamp experiments in the MJF516 strain carrying endogenous MscL. What is the pmsl1/pmscl midpoint (or threshold) ratio? What is the estimated midpoint tension for MSL1?

3. It would be collegial to say that a complete straightening of the pore-lining helices was predicted in the model of fully open MscS and supported by alanine substitutions in positions 113 and 121 (ref. 24). In the A106V crystal structure, these helices are still bent and the pore satisfies approximately 70% of full conductance. It is great to see that in the resolved open state of A320V MSL1 the TM5s are essentially straight and the pore satisfies the full open-state conductance.

4. The boundaries of the membrane anticipated for the closed state imply unnaturally high curvature. Did the authors notice that the nanodisks harboring the same protein are flat? If the authors want to defend the bowl-like shape of the membrane around the channel they should present an estimate of the energetic cost it might entail.

5. The flattening of the barrel in the open state is drastic. The proposed transition drives a lot of protein into the membrane. With this regard it would be necessary to discuss these aspects:
a. Because the authors used carbon grids for all preparations, 2D classes will be dominated by upright orientations, especially for the open state. The external edge of the channel will be adherent to the carbon film surface, which may cause additional artifactual flattening. The authors should explain the contributions of top and side views to the final model.
b. What happens to the electrostatic anchors (R, K) on TM helices in the open state? Are they still within the reach of lipid phosphates?
c. The depicted flattening produces ~23 A^2 increase in the in-plane area associated with opening, which is about twice what is predicted for EcMscS based on the slope of its dose-response. Also, flattening of the presumed cup-shaped segment of the membrane around the channel is expected to produce additional expansion of the system. What area is predicted from the slope of MSL1 ramp responses?

It is understood that it is impossible to do extra experiments under today’s circumstances, but the structure emphasizes the need for the experimental measurement of the expansion area from electrophysiology.


Reviewer #2 (Remarks to the Author):

This structural study of the eukaryotic mechanosensitive channels of small conductance (MscS) from Arabidopsis thaliana describes the open and closed conformations of the channel resolved by cryoEM. The study found similar structures of the channel when it is solubilized in detergents compared to when it is in lipid nanodiscs, providing evidence that detergent-based structures are showing the relevant conformation of the channel. Like previous structural studies of MscS from E. coli, the wild-type channel from Arabidopsis thaliana is in the closed conformation. By using the same mutation strategy that allowed the bacterial channel to be captured in the open conformation, the authors produced a variant channel that allowed for the open channel structure to be elucidated by CryoEM. The authors show that in the open channel conformation, the curved transmembrane domain of MSL1 seen in the closed conformation expands and becomes flattened, akin to the force-induced flattening of Piezo 1 channels into the membrane observed by high-speed atomic force microscopy. The authors propose membrane deformation as a unified mechanical gating mechanism in evolutionarily and architecturally unrelated mechanosensing channels.

The study is well-executed and the data in general supports the conclusions. The open channel structure described for the first time in a eukaryotic mechanosensitive channel is significant. The authors are also commended for validating detergent solubilized structures in nanodiscs. There are however some concerns. The mutation-induced presumed open state may not truly inform structural changes that occur during gating. In addition, there are questions concerning the correlation between electrophysiological and structural data. Below are the detailed comments.

1. Page 4: The authors are cautious in stating they have determined structures of AtMSL1 “presumably” representing the closed and open conformation. This should be consistent in the abstract.
2. Fig.3C shows that AtMSL1 A320V has several subconductance states. It would be helpful to discuss how these subconductance states arise.
3. The authors introduced the A320V mutation to obtain the open channel conformation. This seems to imply that in the structural determination condition, the channel is most stable in the open conformation without added mechanical force. However, in Fig.3C, pressure was applied to open the mutant channel which then stays open for the duration of the recording shown. This indicates in the recording condition, the channel is closed until pressure is applied. The latter does not seem to fit with the statement that a single mutation in the pore-lining helix “induces” channel opening (page 10, line 3). This should be clearly explained.
4. Page 10, Discussion: It is stated that additional helices could function as an extension of the tension sensor. Is there a correlation between the number of TM helices and tension sensitivity? What about the role of lipids? Are plant mitochondrial membrane lipids significantly different from the plasma membrane in eukaryotic cells?
5. As the authors state a major significance of this work is that this is the first eukaryotic MscS structure with both closed and open conformation, they should put MscS from Arabidopsis thaliana into evolutionary context within the MscS family. Specifically, supplementary figure 3 shows a sequence alignment of MscS homologs that are structurally known. It would be helpful to include a percent sequence identity for each of the homologs aligned to allow the divergence between these homologs to be easily seen. Also, as these are sequences for structurally known homologs, a figure showing either an alignment between the structures or a phylogenetic tree could be very useful for putting this Arabidopsis thaliana structure into the context of the family of proteins.
6. The A320V mutant structure in nanodisc only reached 8Å. Can the authors comment on why this is the case?
7. Supplementary figure 4 shows the bacterial and eukaryotic structures side by side. Since both the open and closed conformations are known for both the bacterial and eukaryotic channels, this comparison should include both conformations especially considering that the difference in the movement direction of the tension sensor (page 10, before Discussion).
8. A paper which came out in Cell Rep (Li et al., 30, 4518-4527) while this manuscript is under review presented cryoEM structure of the same channel at 3.3Å. Although the paper only reported the AtMSL1 structure in the closed conformation, it contains more in-depth analysis of the structural and functional differences between AtMSL1 and EcMscS. The authors should cite this paper and note any difference.


Reviewer #3 (Remarks to the Author):

Deng et al report a model for gating of the mechanosensitive ion channel Msl1 from Arabidopsis, based on cryo-EM structures of the wild type channel and a mutant that favors channel opening. The structures are interesting and the work is performed well. I have concerns about interpretation and presentation that should be addressed before publication.

Major points:

1. More consideration needs to be given to what functional states the structures represent. Most importantly, how can the authors reconcile the assignment of an open state to the A320V mutant when it appears to have a similarly low open probability in low tension compared to wild-type and both structures are determined in the same near zero tension conditions?
a. The resting open probability (at low tension without pressure) for each channel should be stated. I assume they are both near zero based on the records shown.
b. A hydrophobic gate is postulated by analogy to previous structures of MscS to for the wild-type channel. It would be useful to include a figure (perhaps from CHAP) to compare the hydrophobicity of the conduction pathway in both Msl1 structures here and E.coli MscS structures. Is the conduction path significantly less hydrophobic in the mutants?
c. The conduction path in the mutant is clearly larger in diameter than in the wild-type. If it is similarly hydrophobic, the authors should be explicit about the models and assumptions they use to conclude that it will be wetted in the mutant and dewetted in the wild-type.
d. The authors tabulate a maximum conductance for the wild-type and mutants channels and say that they are the same. However, it is clear from the record shown that the mutant populates stable subconductance states. Are these observed in the wild-type as well?
e. In E.coli MscS, the analogous mutation results in channels that reach a short-lived open state at low pressure and a long-lived open state only at high pressure. Is this the same observed for Msl1? The conductance for the E. coli MscS mutant is also smaller than wild-type. Based on the structures, do the authors have any explanation for the difference in properties of the two mutants?
f. Are there other populated classes of particles that can be assigned a different conformation in the Msl1 datasets?

2. A more thorough comparison to the E. coli MscS wild-type and analogous A106V structures should be made. It is difficult to assess how different the MscS and Msl1 structures are. Including figures with overlays of each subunit (perhaps aligned in different regions if there are movements of domains with respect to one another) and rmsds would be more useful than the side by side picture in Fig. S4 alone.

3. The mechanism presented in the discussion needs to be elaborated on. The degree of area expansion and the degree of bending/flattening should be quantified from the structures. Does area expansion alone account for the expected deltaG between states?

4. The degree of membrane curvature induced by Piezo channels is much more dramatic than in Msl1. Can the authors provide an analysis of the amount of curvature in Piezos and Msl1 to make clear why a comparison of membrane mending in these channels is helpful?

5. There are already structures of E. coli MscS in a presumed closed (wild-type) and open states (with the analogous mutation). These structures are, as far as I can tell, quite similar to the structures reported here. There does not seem to be a different mechanism at play, rather some of the conformational changes appear to happen to different degrees. That doesn’t make this work less interesting, but it does make this statement in the discussion feel like an overstatement: “In this work we provide, for the first time, a near-atomic visualization of this gating process.” Please rephrase.

Minor points:
1. It would be useful to see figures showing where the lipid belt from the nanodisc is observed in the structures. Is it clearly bent in the wild-type and not in the mutant?

2. On page 8 it states: “All three channels had statistically indistinguishable unitary conductances and tension sensitivities when measured at a membrane potential of -70 mV (Table 2).” However, only pressure was reported. Pressure is not equivalent to tension and unless tension was calculated from images of each patch as well and not reported, this statement should be changed.

**Author rebuttal, first version:**

**Reviewer #1** (Remarks to the Author):

The paper ‘Structural mechanism for gating of a eukaryotic mechanosensitive channel of small conductance’ by Deng and coauthors is a substantial contribution to the field. MSL1 is the first 5-TM representative of the MscS family to be structurally characterized to a resolution that allows for an atomic model to be built for many critical parts of the protein. The important features of the revealed open-state conformation are: (1) the pore-lining helices are tilted and straight, such that the width of the open pore is now sufficient to account for the experimental conductance; and (2) there is a tight contact between the lipid-facing helices and the pore-lining TM5s that furnishes a force-transmission route from the lipid bilayer to the gate. These features were discussed in the field in the form of models, but now for the first time, they are explicitly seen in an open channel structure. The paper reads well and the graphical material is clear and adequate. The following information needs to be included.

**Point 1**: Because EcMscS inactivates (refs 14, 26), and the initial crystal structure (2OAU) was proposed to represent this functional state (ref 24), the authors should say whether or not inactivation was observed in MSL1 under any experimental circumstances (tension, voltage, crowders, etc). This is not a random question because MSL1 fulfills its function under high voltage (150-180 mV) in the inner membrane of an energized mitochondrion. A single MSL1 opening will completely uncouple the energetics and for this reason, a ‘safe’ tension-insensitive and impermeable state might exist. If indeed MSL1 shows the ability to inactivate, it should be discussed if the state with uncoupled TM4 and TM5 helices is closed or inactivated.

**Response:** This is indeed an important question but is challenging to address with our current expression system for *At*MSL1. Given the low number of *At*MSL1 channel activities per patch (1-3 channels, typically), it would be difficult to detect, and even more challenging to distinguish between inactivation and desensitization. We note that inactivation was not reported in either of two published papers on MSL1 (Lee et al., 2016; Li et al., 2020). In this study, we also did not observe any loss of channel activity with repeated ramps applied to a given patch. To address this in the manuscript, we have now added the following (lines 184-186):

The reviewer raises a very good point about the existence of a ‘safe’ impermeable state of MSL1 in mitochondria. Unfortunately, due to the lack of experimental data we can only speculate on the nature of this state and the mechanisms bringing the channel into it. One of the possibilities is fast negative feedback triggered by membrane depolarization, implying blocking or inhibiting the channel via direct interaction with certain molecule(s). Another possibility is modulation of gating threshold by intra-mitochondrial crowders, similar to *Ec*MscS (Rowe et al., 2014). Indeed, a high apparent viscosity of mitochondrial matrix resulting from high protein and metabolite content (Soboll et al., 1976, Tischler et al., 1977) and compartmentalization have been measured by a number of physical methods (Dieteren et al., 2011; Chambers et al., 2018).

“*However, unlike EcMscS^14,26^, neither AtMSL1 nor AtMSL1 A320V displayed inactivation in response to repeated pressure ramps, regardless of ramp rate or length*.”

*References:*

1. Lee CP, Maksaev G, Jensen GS, Murcha MW, Wilson ME, Fricker M, Hell R, Haswell ES, Millar AH, Sweetlove LJ. MSL1 is a mechanosensitive ion channel that dissipates mitochondrial membrane potential and maintains redox homeostasis in mitochondria during abiotic stress. *Plant J* 88:809-825 (2016).
2. Li Y, Hu Y, Wang J, Liu X, Zhang W, Sun L. Structural Insights into a Plant Mechanosensitive Ion Channel MSL1. *Cell Rep* 30:4518-4527.e3 (2020).
3. Rowe I, Anishkin A, Kamaraju K, Yoshimura K, Sukharev S. The cytoplasmic cage domain of

the mechanosensitive channel MscS is a sensor of macromolecular crowding. *J Gen Physiol* 143:543-57 (2014).

4. Soboll S, Scholz R, Freisl M, Elbers R, Heldt HW. In: Use of Isolated Liver Cells and Kidney Tubules in Metabolic Studies (Tager JM et al eds): 29-40. Academic Press, London, New York (1976).
5. Tischler ME, Hecht P, Williamson JR. Determination of Mitochondrial/Cytosolic Metabolite Gradients in Isolated Rat Liver Cells by Cell Disruption. *Archives of Biochemistry and Biophysics* 181:278-292 (1977).
6. Dieteren CE, Gielen SC, Nijtmans LG, Smeitink JA, Swarts HG, Brock R, Willems PH, Koopman WJ. Solute diffusion is hindered in the mitochondrial matrix. *Proc Natl Acad Sci USA* 108:8657-62 (2011).
7. Chambers JE, Kubánková M, Huber RG, López-Duarte I, Avezov E, Bond PJ, Marciniak SJ, Kuimova MK. An Optical Technique for Mapping Microviscosity Dynamics in Cellular Organelles. *ACS Nano* 12:4398-4407 (2018).

**Point 2:** The authors perform their patch-clamp experiments in the MJF516 strain carrying endogenous MscL. What is the pmsl1/pmscl midpoint (or threshold) ratio? What is the estimated midpoint tension for MSL1?

**Response:** Threshold ratios for gating pressure were presented in Table 2 (now new **Table 3**). These numbers (0.64 ± 0.14 for MSL1 and 0.55 ± 0.15 for the A320V variant) have now also been added to the main text (line 183). Due to technical limitations (low number of channels, patch rupture before saturation), we are unable to calculate the midpoint ratio. We note that our main goal here is to provide support for the interpretation of the structures, not to provide a full characterization of MSL1 channel behavior.

**Point 3:** It would be collegial to say that a complete straightening of the pore-lining helices was predicted in the model of fully open MscS and supported by alanine substitutions in positions 113 and 121 (ref. 24). In the A106V crystal structure, these helices are still bent and the pore satisfies approximately 70% of full conductance. It is great to see that in the resolved open state of A320V MSL1 the TM5s are essentially straight and the pore satisfies the full open-state conductance.

**Response:** We thank the reviewer for this point and have now added the following in the main text (lines 219-221):

**Point 4:** The boundaries of the membrane anticipated for the closed state imply unnaturally high curvature. Did the authors notice that the nanodisks harboring the same protein are flat? If the authors want to defend the bowl-like shape of the membrane around the channel they should present an estimate of the energetic cost it might entail.

“*Consistently, straightening of the pore-lining helix was suggested in a simulated open EcMscS conformation, which was further supported by patch-clamp experiments of alanine substitutions at positions near the kink^24^*.”

**Response:** Our cryo-EM density maps do not allow us to unambiguously visualize the boundaries of detergent micelles or lipid nanodiscs and therefore we are unable to use these data to quantify the extent of membrane deformations. However, as shown in **Supplementary Fig. 7**, comparison of the transmembrane regions of the closed, bowl-shaped, wild-type and open, flattened A320V channels clearly indicates that membrane surrounding the wild type is

curved but surrounding A320V is flat. Importantly, **Supplementary Fig. 7** also shows that both wild-type and A320V channels maintain their transmembrane domain shapes both in detergent micelles and in lipid nanodiscs. This would be in agreement with the localization of *At*MSL1 in the inner mitochondrial membranes, which form highly curved cristae. Thus it seems plausible that *At*MSL1 might localize to these curved membrane regions.

Because we cannot quantify the extent of membrane deformations, it would be premature to calculate the energetic cost arising from membrane bending. We used distribution of charged residues in the transmembrane region to imply the approximate membrane boundaries in **Fig. 2a**. It is not our intention to claim that this is how much the membrane is curved. We have now removed the illustrated membrane boundaries in **Fig. 2a** to be clear.

5. The flattening of the barrel in the open state is drastic. The proposed transition drives a lot of protein into the membrane. With this regard it would be necessary to discuss these aspects:

**Point 5a:** Because the authors used carbon grids for all preparations, 2D classes will be dominated by upright orientations, especially for the open state. The external edge of the channel will be adherent to the carbon film surface, which may cause additional artifactual flattening. The authors should explain the contributions of top and side views to the final model.

**Response:** For cryo-EM studies, we used copper Quantifoil R2/2 holey carbon grids (Quantifoil), which have holes in the grids. The channel proteins were embedded in vitreous ice formed in the holes and particles in these areas were imaged. Channel proteins were not on

**b	c**

carbon film surface. Inspection of 2D classes indicated random orientations of channel proteins

TI5s	TI5

in vitreous ice. In addition, more quantitatively, the orientation distribution plots of particles used

TI

for final cryo-EM reconstructions (**Supplementary Figs. 1b**, **5b**, and **6b**) showed that there were no preferred orientation issues. These details were included in *Methods* or *Supplementary Figs*.

**Point 5b:** What happens to the electrostatic anchors (R, K) on TM helices in the open state? Are they still within the reach of lipid phosphates?

**Response:** This is a good point. These electrostatic anchors should be within the reach of lipid head groups. We have now included an additional figure (new **Supplementary Fig. 8f**) to

**e	f**

illustrate these charged residues in the transmembrane helices in the open structure.

new **Supplementary Fig. 8f**

**Point 5c:** The depicted flattening produces ~23 A^2 increase in the in-plane area associated with opening, which is about twice what is predicted for EcMscS based on the slope of its dose-

response. Also, flattening of the presumed cup-shaped segment of the membrane around the channel is expected to produce additional expansion of the system. What area is predicted from the slope of MSL1 ramp responses?

It is understood that it is impossible to do extra experiments under today’s circumstances, but the structure emphasizes the need for the experimental measurement of the expansion area from electrophysiology.

**Response:** This is a very good point. However, calculations of in-plane area changes based on electrophysiological data (the slope of MSL1 ramp responses) involve examining a population of channels within a patch and obtaining current saturation (Anishkin et al., 2005; Boer et al., 2011; Maksaev et al., 2011; Sukharev, 2002). This experiment is unfortunately not possible with our current expression system due to a small number of channels (1-3 channels) per patch, as described above.

*Reference*:

1. Sukharev S. Purification of the small mechanosensitive channel of Escherichia coli (MscS): the subunit structure, conduction, and gating characteristics in liposomes. *Biophys J* 83:290-298 (2002).
2. Anishkin A, Chiang CS, Sukharev S. Gain-of-function mutations reveal expanded

intermediate states and a sequential action of two gates in MscL. *J Gen Physiol* 125:155-70 (2005).

3. Boer M, Anishkin A, Sukharev S. Adaptive MscS gating in the osmotic permeability response in E. coli: the question of time. *Biochemistry* 50:4087-96 (2011).
4. Maksaev G, Milac A, Anishkin A, Guy HR, Sukharev S. Analyses of gating thermodynamics and effects of deletions in the mechanosensitive channel TREK-1: comparisons with structural models. *Channels* 5:34-42 (2011).

**Reviewer #2** (Remarks to the Author):

This structural study of the eukaryotic mechanosensitive channels of small conductance (MscS) from Arabidopsis thaliana describes the open and closed conformations of the channel resolved by cryoEM. The study found similar structures of the channel when it is solubilized in detergents compared to when it is in lipid nanodiscs, providing evidence that detergent-based structures are showing the relevant conformation of the channel. Like previous structural studies of MscS from E. coli, the wild-type channel from Arabidopsis thaliana is in the closed conformation. By using the same mutation strategy that allowed the bacterial channel to be captured in the open conformation, the authors produced a variant channel that allowed for the open channel structure to be elucidated by CryoEM. The authors show that in the open channel conformation, the curved transmembrane domain of MSL1 seen in the closed conformation expands and becomes flattened, akin to the force-induced flattening of Piezo1 channels into the membrane observed by high-speed atomic force microscopy. The authors propose membrane deformation as a unified mechanical gating mechanism in evolutionarily and architecturally unrelated mechanosensing channels.

The study is well-executed and the data in general supports the conclusions. The open channel structure described for the first time in a eukaryotic mechanosensitive channel is significant. The authors are also commended for validating detergent solubilized structures in nanodiscs. There are however some concerns. The mutation-induced presumed open state may not truly inform structural changes that occur during gating. In addition, there are questions concerning the correlation between electrophysiological and structural data. Below are the detailed comments.

**Point 1:** Page 4: The authors are cautious in stating they have determined structures of AtMSL1 “presumably” representing the closed and open conformation. This should be consistent in the abstract.

**Response:** We thank the reviewer for this point and have now added “presumably” in the abstract (Line 31-33).

“*Here, we present cryo-electron microscopy structures of a MscS homolog from Arabidopsis thaliana, MSL1, presumably in both the closed and open states*.”

**Point 2:** Fig.3C shows that AtMSL1 A320V has several subconductance states. It would be helpful to discuss how these subconductance states arise.

**Response:** This is an interesting point. In fact, both *At*MSL1 and *At*MSL1 A320V display subconducting states that typically arise after the channel fully opens, although *At*MSL1 A320V enters these subconducting states far more often than *At*MSL1. We now have quantified this effect, put the results in the new **Table 2**, and describe them in the *Results* (lines 178-179):

“*Both AtMSL1 and AtMSL1 A320V exhibited subconducting states, but this was more frequently observed with AtMSL1 A320V (Table 2, Fig. 3c).”*

**Table 2 Spontaneous opening and subconducting states for *At*MSL1 and A320V**. Channel activity prior to pressure ramp application, N = 12-14 patches each. The presence of subconducting states, and if they followed a full channel opening, in lifetime traces of pressure-gated channels only, N = 10 patches for each variant.

*At*MSL1	*At*MSL1 A320V

| Channel(s) open before pressure ramp | 25% | (3/12 traces) | 57% | (8/14 traces) |
| --- | --- | --- | --- | --- |
| Channel exhibits subconducting state | 29% | (30/102 traces) | 50% | (17/34 traces) |
| Subconducting state follows full opening | 17% | (5/30 traces) | 82% | (14/17 traces) |

**Point 3:** The authors introduced the A320V mutation to obtain the open channel conformation. This seems to imply that in the structural determination condition, the channel is most stable in the open conformation without added mechanical force. However, in Fig.3C, pressure was applied to open the mutant channel which then stays open for the duration of the recording shown. This indicates in the recording condition, the channel is closed until pressure is applied. The latter does not seem to fit with the statement that a single mutation in the pore-lining helix “induces” channel opening (page 10, line 3). This should be clearly explained.

**Response:** Gating events in the absence of pressure have been previously noted for *At*MSL1 (Lee et al., 2016) and were observed in our experiments at an even higher rate for *At*MSL1 A320V (57% for A320V compared to 25% for the wild type, please see new **Table 2**). However, the majority of channel activity observed was pressure-dependent. To further clarify, we have changed the phrase “*a single mutation in the pore-lining helix induces channel opening*” to “*a single mutation in the pore-lining helix facilitates channel opening*” (line 226).

*Reference*:

1. Lee CP, Maksaev G, Jensen GS, Murcha MW, Wilson ME, Fricker M, Hell R, Haswell ES, Millar AH, Sweetlove LJ. MSL1 is a mechanosensitive ion channel that dissipates mitochondrial membrane potential and maintains redox homeostasis in mitochondria during abiotic stress.

*Plant J* 88:809-825 (2016).

**Point 4:** Page 10, Discussion: It is stated that additional helices could function as an extension of the tension sensor. Is there a correlation between the number of TM helices and tension sensitivity? What about the role of lipids? Are plant mitochondrial membrane lipids significantly different from the plasma membrane in eukaryotic cells?

**Response:** There is no simple relationship between the number of TM helices and tension sensitivity for members of the MscS family of ion channels. For example, *Ec*MscS, *At*MSL1, and *Ec*YbiO have 3, 5, and 11 TM helices respectively, but all have similar tension sensitivities.

It is likely that lipids play a role in the gating of certain MS channels (Cox et al., 2019), and lipid composition does vary between plant mitochondrial and animal plasma membranes (Horvath & Daum, 2013; Jouhet et al., 2004; Michaud et al., 2016; Pan & Hu, 2015; Pineau et al., 2013).

*References*:

1, Cox CD, Gottlieb PA. Amphipathic molecules modulate PIEZO1 activity. *Biochem Soc Trans* 47:1833-1842 (2019).
2, Horvath SE, Daum G. Lipids of mitochondria. *Prog Lipid Res* 52:590-614 (2013).
3, Jouhet J, Maréchal E, Baldan B, Bligny R, Joyard J, Block MA. Phosphate deprivation induces transfer of DGDG galactolipid from chloroplast to mitochondria. *J Cell Biol* 167:863-74 (2004).
4, Michaud M, Gros V, Tardif M, Brugière S, Ferro M, Prinz WA, Toulmay A, Mathur J, Wozny M, Falconet D, Maréchal E, Block MA, Jouhet J. AtMic60 Is Involved in Plant Mitochondria Lipid Trafficking and Is Part of a Large Complex. *Curr Biol* 26:627-39 (2016).
5, Pan R, Hu J. Plant mitochondrial dynamics and the role of membrane lipids. *Plant Signal Behav* 10:e1050573 (2015).
6, Pineau B, Bourge M, Marion J, Mauve C, Gilard F, Maneta-Peyret L, Moreau P, Satiat-Jeunemaître B, Brown SC, De Paepe R, Danon A. The importance of cardiolipin synthase for mitochondrial ultrastructure, respiratory function, plant development, and stress responses in Arabidopsis. *Plant Cell* 25:4195-208 (2013).

**Point 5:** As the authors state a major significance of this work is that this is the first eukaryotic MscS structure with both closed and open conformation, they should put MscS from Arabidopsis thaliana into evolutionary context within the MscS family. Specifically, supplementary figure 3 shows a sequence alignment of MscS homologs that are structurally known. It would be helpful to include a percent sequence identity for each of the homologs aligned to allow the divergence between these homologs to be easily seen. Also, as these are sequences for structurally known homologs, a figure showing either an alignment between the structures or a phylogenetic tree could be very useful for putting this Arabidopsis thaliana structure into the context of the family of proteins.

**Response:** We appreciate this good point and have now included a phylogenetic tree with sequence identity in new **Supplementary Fig. 3b**.

*Hp*MscS 17.9%

b

*Ec*MscS 18.2%

*Tt*MscS 14.9% *At*MSL1 100% *Ec*YnaI 24.8%

new **Supplementary Fig. 3b**

**Point 6:** The A320V mutant structure in nanodisc only reached 8Å. Can the authors comment on why this is the case?

**Response:** This is an interesting point but we do not fully understand why. It often happens that lipid nanodisc reconstitutions of membrane proteins do not yield high resolution structures. We noticed that during preparation of A320V-nanodiscs, samples were biochemically less stable than A320V-micelles or wild-type-nanodiscs. Perhaps the limited dimension of nanodiscs is too tight for the open A320V structure with an expanded transmembrane domain.

**Point 7:** Supplementary figure 4 shows the bacterial and eukaryotic structures side by side. Since both the open and closed conformations are known for both the bacterial and eukaryotic channels, this comparison should include both conformations especially considering that the difference in the movement direction of the tension sensor (page 10, before Discussion).

**Response:** This is a good point and we have now included a new **Supplementary Fig. 9** to compare all these structures side by side.

**a**	Closed MSL1	**b**	Closed MscS

**“*Our structure is further corroborated by a recent independent cryo-EM structure of AtMSL1 that was expressed in mammalian cells^29^. The root-mean-square deviation (r.m.s.d) between***

new **Supplementary Fig. 9**

Open MscS

**c	d**

Open MSL1

**e	f**

**g**

Opening	Opening

**Point 8:** A paper which came out in Cell Rep (Li et al., 30, 4518-4527) while this manuscript is under review presented cryoEM structure of the same channel at 3.3Å. Although the paper only reported the AtMSL1 structure in the closed conformation, it contains more in-depth analysis of the structural and functional differences between AtMSL1 and EcMscS. The authors should cite this paper and note any difference.

**Response:** We have now explicitly cited the new *Cell Rep* paper and added the following paragraph (lines 121-126).

**Reviewer #3** (Remarks to the Author):

***these two structures is ~1.4 Å for Cα atoms from TM4 to the C-terminus. The most pronounced differences arise from the peripheral TM2 and TM3 helices that appear to be dynamic owing to lack of protein-protein interactions within the heptameric channel (r.m.s.d ~3.1 Å for Cα atoms in TM2 and TM3).*”**

Deng et al report a model for gating of the mechanosensitive ion channel Msl1 from Arabidopsis, based on cryo-EM structures of the wild type channel and a mutant that favors channel opening. The structures are interesting and the work is performed well. I have concerns about interpretation and presentation that should be addressed before publication.

Major points:

**Point 1:** More consideration needs to be given to what functional states the structures represent. Most importantly, how can the authors reconcile the assignment of an open state to the A320V mutant when it appears to have a similarly low open probability in low tension compared to wild-type and both structures are determined in the same near zero tension conditions?

**Response:** The assignment of an open state to the *At*MSL1 A320V structure is based on the following observations:

1. The A320V mutant channels in excised patches open spontaneously more often than the wild-type MSL1 prior to pressure ramps (new **Table 2**).
2. The conduction pore of A320V is much wider than that of the wild type (**Fig. 4d**) and is water-accessible, while the wild type is not (new **Supplementary Fig. 9**, also see **Response** to **Point 1b**,**1c** below). In addition, the A320V pore is wider than the ‘open’ pore of *Ec*MscS A106V (PDB: 2VV5) (**Fig. 2c**, **4d**, and new **Supplementary Fig. 9c,d**).
3. Consistently, A320V has a much increased in-plane area in the transmembrane region compared with the wild type.

Therefore, we think that it is reasonable to suggest that the A320V structure represents an open conformation.

**Point 1a:** The resting open probability (at low tension without pressure) for each channel should be stated. I assume they are both near zero based on the records shown.

**Response:** Unfortunately, the low number of gating events per patch and/or our inability to achieve saturation prevent us from making a reasonable estimation of the resting open probability for either version of *At*MSL1. However, we have added a new table that reports the number of times that channel opening was observed prior to the application of pressure (new **Table 2**), which we hope addresses the same point. This is now described in *Results* (lines 179­181):

**Table 2 Spontaneous opening and subconducting states for *At*MSL1 and A320V**. Channel activity prior to pressure ramp application, N = 12-14 patches each. The presence of subconducting states, and if they followed a full channel opening, in lifetime traces of pressure-gated channels only, N = 10 patches for each variant.

“*Furthermore, AtMSL1 A320V was more likely than AtMSL1 to open immediately after patch formation and prior to the application of pressure ramps (Table 2)*.”

*At*MSL1	*At*MSL1 A320V

| Channel(s) open before pressure ramp | 25% | (3/12 traces) | 57% | (8/14 traces) |
| --- | --- | --- | --- | --- |
| Channel exhibits subconducting state | 29% | (30/102 traces) | 50% | (17/34 traces) |
| Subconducting state follows full opening | 17% | (5/30 traces) | 82% | (14/17 traces) |

**Point 1b**: A hydrophobic gate is postulated by analogy to previous structures of MscS to for the wild-type channel. It would be useful to include a figure (perhaps from CHAP) to compare the hydrophobicity of the conduction pathway in both Msl1 structures here and E.coli MscS structures. Is the conduction path significantly less hydrophobic in the mutants?

**Response:** We thank the reviewer for this point and have now included new **Supplementary Fig. 9** to compare hydrophobicity of the conduction pathway in MSL1 and *Ec*MscS structures. Indeed the conduction path is significantly less hydrophobic in the A320V open structure, especially for the ‘hydrophobic gate’ region (Red: more hydrophobic; white: less hydrophobic).

new **Supplementary Fig. 9**

a	Closed MSL1	b	Closed MscS

Open MscS

c	d

Open MSL1

f

**Point 1c:** The conduction path in the mutant is clearly larger in diameter than in the wild-type. If it is similarly hydrophobic, the authors should be explicit about the models and assumptions they use to conclude that it will be wetted in the mutant and dewetted in the wild-type.

**Response:** We appreciate this point. The new **Supplementary Fig. 9** shows that the conduction path becomes less hydrophobic at the narrow pore region as the pore dilates. This further suggests that the A320V structure represents an open, conductive conformation. We have now added the following in the revised manuscript (lines 214-219):

“*Concomitantly, the outward movement of the straightened TM5 helix results in wide opening of the hydrophobic gate (Fig. 4h, Supplementary Fig. 8), which renders the conduction path less hydrophobic at this region (Supplementary Fig. 9). The increased pore diameter and decreased hydrophobicity support our conclusion that the A320V structure represents an open, conductive conformation*.”

**Point 1d:** The authors tabulate a maximum conductance for the wild-type and mutants channels and say that they are the same. However, it is clear from the record shown that the mutant populates stable subconductance states. Are these observed in the wild-type as well?

**Response:** Please also see **Response** to **Point 2** of **Reviewer #2**.

Both *At*MSL1 and *At*MSL1 A320V display subconducting states that typically arise after the channel fully opens, although *At*MSL1 A320V enters these subconducting states far more often than *At*MSL1. We now have quantified this effect, put the results in new **Table 2**, and describe them in the *Results* (lines 178-179):

**Table 2 Spontaneous opening and subconducting states for *At*MSL1 and A320V**. Channel activity prior to pressure ramp application, N = 12-14 patches each. The presence of subconducting states, and if they followed a full channel opening, in lifetime traces of pressure-gated channels only, N = 10 patches for each variant.

“*Both AtMSL1 and AtMSL1 A320V exhibited subconducting states, but this was more frequently observed with AtMSL1 A320V (Table 2, Fig. 3c)*.”

*At*MSL1	*At*MSL1 A320V

| Channel(s) open before pressure ramp | 25% | (3/12 traces) | 57% | (8/14 traces) |
| --- | --- | --- | --- | --- |
| Channel exhibits subconducting state | 29% | (30/102 traces) | 50% | (17/34 traces) |
| Subconducting state follows full opening | 17% | (5/30 traces) | 82% | (14/17 traces) |

**Point 1e:** In E.coli MscS, the analogous mutation results in channels that reach a short-lived open state at low pressure and a long-lived open state only at high pressure. Is this the same observed for Msl1? The conductance for the E. coli MscS mutant is also smaller than wild-type. Based on the structures, do the authors have any explanation for the difference in properties of the two mutants?

**Response:** These properties in E. coli MscS were not observed in *At*MSL1. **Fig. 2c** and new **Supplementary Fig. 9** indicate that the pore of the wild-type *At*MSL1 is approximately as narrow as that of the wild-type *Ec*MscS (-4 Å radius at the narrowest constriction). However, the pore of the *At*MSL1 A320V is much wider than that of the equivalent *Ec*MscS A106V (-8 Å

vs ~6 Å radius at the narrowest, **Fig. 2c**, **4d** and new **Supplementary Fig. 9**). This would be consistent with our suggestion that the *At*MSL1 A320V structure represents an open conformation and that the unitary conductance of *At*MSL1 A320V could be similar to those of the wild-type *At*MSL1 and wild-type *Ec*MscS.

**Point 1f:** Are there other populated classes of particles that can be assigned a different conformation in the Msl1 datasets?

**Response:** In all MSL1 datasets, inspection of 3D classifications indicated that there were no

β barrel

other populated classes of particles that could be assigned a different conformation.

**c	d**

**Point 2:** A more thorough comparison to the E. coli MscS wild-type and analogous A106V structures should be made. It is difficult to assess how different the MscS and Msl1 structures are. Including figures with overlays of each subunit (perhaps aligned in different regions if there are movements of domains with respect to one another) and rmsds would be more useful than the side by side picture in Fig. S4 alone.

**Response:** In our revised manuscript, we have now included overlays of wild-type *At*MSL1 and wild-type *Ec*MscS (new **Supplementary Fig. 4g**), and the open mutants *At*MSL1 A320V and *Ec*MscS A106V (new **Supplementary Fig. 9g**). Information for the alignment and rmsd of Cα

Side
portal

are included in the figure legends.

new **Supplementary Fig. 9g**

new **Supplementary Fig. 4g**

**g**

**e	f**

Opening	Opening

**e	f**

**g**

**Point 3:** The mechanism presented in the discussion needs to be elaborated on. The degree of area expansion and the degree of bending/flattening should be quantified from the structures.

Does area expansion alone account for the expected deltaG between states?

**Response:** This is a good point but our experimental limitations prevented us from being more quantitative. The in-plane area expansion of the transmembrane region is ~23 nm^2^ for resolved TM2-TM5 helices. This has now been added in *Discussion* (line 249). Both area expansion and bending will contribute to gating. We have modified the equation to explicitly clarify this point (lines 241-243).

*∆ = (∆ _channel_ + ∆ bending) − ∆*

Our cryo-EM density maps do not allow us to unambiguously visualize the boundaries of detergent micelles or lipid nanodiscs and therefore we are unable to use these data to quantify the extent of membrane bending associated with the closed and open conformations of MSL1.

where *∆!!!""#$* is the difference of free energy intrinsic to channel gating, and *_∆bend%n&_* the

free energy difference of membrane bending associated with channel gating.

An experimental approach to measure these terms is to use the dose-response activation curve

for Boltzmann fitting, from which LG ( *∆!!!""#$* + *_∆bending_*_),_ a (membrane tension) and LA (in-plane area expansion) could be derived: Po = 1 / (1+exp(LG-aLA)/kT) (as it was done, for example, in Belyy et al., *J Gen Physiol* 2010). Unfortunately, the number of MSL1 channels per patch in our hands was typically between 1 and 3, which prevented us from plotting dose-response curves. Therefore, a quantitative interpretation of this gating mechanism (equation) is beyond our experimental capability and out of the scope of our current study.

*Reference*:

1. Belyy V, Kamaraju K, Akitake B, Anishkin A, Sukharev S. Adaptive Behavior of Bacterial Mechanosensitive Channels is Coupled to Membrane Mechanics. *J Gen Physiol* 135:641-652 (2010).

**Point 4:** The degree of membrane curvature induced by Piezo channels is much more dramatic than in Msl1. Can the authors provide an analysis of the amount of curvature in Piezos and Msl1 to make clear why a comparison of membrane mending in these channels is helpful?

**Response:** We agree that membrane curvature induced by Piezo channels is much more dramatic as these channels have very long transmembrane arms. Thorough analysis of Piezo1-related membrane deformations was elegantly presented elsewhere [Guo & MacKinnon, eLife 2017; Haselwandter & MacKinnon, eLife 2018]. We could not provide a quantitative analysis of membrane curvature as reasons elaborated above (also see **Response** to **Point 3**). However, membrane curvature observed in MSL1, clearly indicated by the bowl-shaped, closed and flattened, open structures, is also quite dramatic with respect to a much smaller channel in size.

*Reference*:

1. Guo YR, MacKinnon R. Structure-based Membrane Dome Mechanism for Piezo Mechanosensitivity. *eLife* 6:e33660 (2017).
2. Haselwandter CA, MacKinnon R. Piezo’s Membrane Footprint and Its Contribution to Mechanosensitivity. *eLife* 7:e41968 (2018).

**Point 5:** There are already structures of E. coli MscS in a presumed closed (wild-type) and open states (with the analogous mutation). These structures are, as far as I can tell, quite similar to

the structures reported here. There does not seem to be a different mechanism at play, rather some of the conformational changes appear to happen to different degrees. That doesn’t make this work less interesting, but it does make this statement in the discussion feel like an overstatement: “In this work we provide, for the first time, a near-atomic visualization of this gating process.” Please rephrase.

**Response:** As illustrated in **Supplementary Movies 1** and **2** and new **Supplementary Fig. 9**, the opening transitions of *Ec*MscS and *At*MSL1 are quite different. In particular, an obvious “flattened” transmembrane region upon opening of *At*MSL1 is indeed the first observation at a near-atomic level (which is not apparent in *Ec*MscS gating). This point is also noted by **Reviewer #1** stating “*These features were discussed in the field in the form of models, but now for the first time, they are explicitly seen in an open channel structure*”. To be more clear on this point, we rephrased this statement (line 255-257).

“*In this work we provide, for the first time, a near-atomic visualization of the flattening and expansion process involved in channel gating*.”

Minor points:

**Point 1:** It would be useful to see figures showing where the lipid belt from the nanodisc is

observed in the structures. Is it clearly bent in the wild-type and not in the mutant?

**Response:** Our cryo-EM density maps do not allow us to unambiguously visualize the boundaries of detergent micelles or lipid nanodiscs. Membrane deformation is implied by the curved transmembrane domains of the wild-type channel.

**Point 2:** On page 8 it states: “All three channels had statistically indistinguishable unitary conductances and tension sensitivities when measured at a membrane potential of -70 mV (Table 2).” However, only pressure was reported. Pressure is not equivalent to tension and unless tension was calculated from images of each patch as well and not reported, this statement should be changed.

**Response:** This is correct. The text has been changed to state that relative gating pressures , not tension sensitivities, were indistinguishable (line 182).

**Reviewer comments, second version:**

Reviewer #1 (Remarks to the Author):

I am generally satisfied with the revisions.
Please consider the following suggestions that may improve readability.

The Piezo channel outline is wider and can be called ‘bowl-shaped’. AtMSL1 is considerably smaller and implies a more curved membrane. Wouldn’t it be better to call it ‘cup-shaped’?

L 163-169: Please separate the sentences that describe the strategic position of A106 and homologous A320 in two different species. It is hard to read.

L179-181: AtMSL1 A320V frequently opens spontaneously upon seal formation. It might be good to mention that resting tension in patches always exists due to membrane-glass adhesion forces.

L182-183: and relative gating pressure thresholds (0.49…)

L 184-187: You stated that under your experimental conditions there were signs of inactivation in neither AtMSL1 nor in the mutant. The hallmark of EcMscS, however, is that it is a 3-state channel. Would it be reasonable to say in the Discussion that a search for inactivation conditions for AtMSL1 is warranted?

L210: …as a rigid body by a rotation of ~135o and… Rotation about what axis?

L231-233: You need to better explain what structural part is assigned the function of the ‘tension sensor’ in each channel.

L256: The term ‘flattening’ appears in two different connotations. There is a flattening of the barrel structure (reduction in height) and there is alleged flattening of the cup-shaped membrane segment embracing the protein. Please make an explicit distinction between the two objects and transitions in the text.


Reviewer #2 (Remarks to the Author):

The authors have made satisfactory revision to address reviewer concerns.


Reviewer #3 (Remarks to the Author):

I am satisfied with the authors response to the reviews.

I only suggest, at the authors discretion, they consider whether they could make an estimation of resting open probability from their electrophysiological data in hand. It would seem possible, given the 12 and 14 records for AtMSL1 and the A320V mutant analyzed for openings before pressure (Table 2), to calculate a Po for each patch before pressure and ask whether the means across patches are different for wild type and mutant. With one to three channels per patch, even if it unclear exactly how many channels there are, one could make an estimate assuming either one (for a maximum resting Po)and three channels (for a minimum resting Po) per patch. If there are clear differences as one might expect from Table 2, it would further support the interpretation of the structures.

**Author rebuttal, second version:**

Reviewer #1 (Remarks to the Author):

I am generally satisfied with the revisions.

Please consider the following suggestions that may improve readability.

**Point 1:** The Piezo channel outline is wider and can be called ‘bowl-shaped’. AtMSL1 is considerably smaller and implies a more curved membrane. Wouldn’t it be better to call it ‘cup-shaped’?

**Response:** We would like to keep ‘bowl-shaped’ as ‘bowl’ can have different size and curvature.

**Point 2:** L 163-169: Please separate the sentences that describe the strategic position of A106 and homologous A320 in two different species. It is hard to read.

**Response:** The sentences have now been simplified in new Lines L170-173.

“*This position, strategically located between two consecutive pore narrowing residues (L105 and L109), is highly conserved among MscS homologs and appears to be essential for the tight helix packing that stabilizes the closed conformation (Fig. 3a, b). Inspired by these observations, we introduced the equivalent mutation in AtMSL1 (A320V) to evaluate whether analogous destabilization accordingly favors an open conformation*.”

**Point 3:** L179-181: AtMSL1 A320V frequently opens spontaneously upon seal formation. It might be good to mention that resting tension in patches always exists due to membrane-glass adhesion forces.

**Response:** We have now included this point. New lines 187-188:

**Point 4:** L182-183: and relative gating pressure thresholds (0.49...) **Response:** Thanks and we added ‘*thresholds*’ (Line 190).

“*as the patch of membrane within the glass pipette has inherent lateral tension due to the lipid-glass adhesion force*.”

**Point 5:** L 184-187: You stated that under your experimental conditions there were signs of inactivation in neither AtMSL1 nor in the mutant. The hallmark of EcMscS, however, is that it is a 3-state channel. Would it be reasonable to say in the Discussion that a search for inactivation conditions for AtMSL1 is warranted?

**Response:** Since we do not have any insights into AtMSL1 inactivation, we would like to stay away from speculating on this point in the Discussion.

**Point 6:** L210: ...as a rigid body by a rotation of ~135o and... Rotation about what axis?

**Response:** We have now added the information in Line 221: “(*approximately about the helical axis of TM3*)”.

**Point 7:** L231-233: You need to better explain what structural part is assigned the function of the ‘tension sensor’ in each channel.

**Response:** We have now explicitly defined the structural part assigned to the ‘tension sensor’ in each channel (Line 244).

“...*possess a peripheral ‘tension sensor’ (TM1-2 in EcMscS and TM1-4 in AtMSL1)*...”

**Point 8:** L256: The term ‘flattening’ appears in two different connotations. There is a flattening of the barrel structure (reduction in height) and there is alleged flattening of the cup-shaped membrane segment embracing the protein. Please make an explicit distinction between the two objects and transitions in the text.

**Response:** We have now explicitly stated: “...the membrane flattening...” (Line 268).

Reviewer #2 (Remarks to the Author):

The authors have made satisfactory revision to address reviewer concerns.

Reviewer #3 (Remarks to the Author):

I am satisfied with the authors response to the reviews.

**Point 1:** I only suggest, at the authors discretion, they consider whether they could make an estimation of resting open probability from their electrophysiological data in hand. It would seem possible, given the 12 and 14 records for AtMSL1 and the A320V mutant analyzed for openings before pressure (Table 2), to calculate a Po for each patch before pressure and ask whether the means across patches are different for wild type and mutant. With one to three channels per patch, even if it unclear exactly how many channels there are, one could make an estimate assuming either one (for a maximum resting Po)and three channels (for a minimum resting Po) per patch. If there are clear differences as one might expect from Table 2, it would further support the interpretation of the structures.

**Response:** We thank the reviewer for this point. We have considered this but would like to leave it as is.

**Reviewer comments, third version:**

**Author rebuttal, third version:**
